# Traditional Uses, Phytochemistry and Biological Activities of *Alocasia* Species: A Systematic Review

**DOI:** 10.3389/fphar.2022.849704

**Published:** 2022-05-24

**Authors:** Dayar Arbain, Lorenskia Maria Regina Sinaga, Muhammad Taher, Deny Susanti, Zainul Amiruddin Zakaria, Junaidi Khotib

**Affiliations:** ^1^ Faculty of Pharmacy, Universitas 17 Agustus 1945, Jakarta, Indonesia; ^2^ Department of Pharmaceutical Technology, Kulliyyah of Pharmacy, International Islamic University Malaysia, Kuantan, Malaysia; ^3^ Pharmaceutics and Translational Research Group, Kulliyyah of Pharmacy, International Islamic University Malaysia, Kuantan, Malaysia; ^4^ Department of Chemistry, Kulliyyah of Science, International Islamic University Malaysia, Kuantan, Malaysia; ^5^ Department of Biomedical Sciences, Faculty of Medical and Health Sciences, Universiti Malaysia Sabah, Kota Kinabalu, Malaysia; ^6^ Department of Pharmacy Practice, Faculty of Pharmacy, Airlangga University, Surabaya, Indonesia

**Keywords:** Alocasia, keladi liar, talas liar, bioactivities, giant taro

## Abstract

The genus *Alocasia* (Schott) G. Don consists of 113 species distributed across Asia, Southeast Asia, and Australia. *Alocasia* plants grow in tropical and subtropical forests with humid lowlands. Featuring their large green heart-shaped or arrow-shaped ear leaves and occasionally red-orange fruit, they are very popular ornamental plants and are widely used as traditional medicines to treat various diseases such as jaundice, snake bite, boils, and diabetes. This manuscript critically analysed the distribution, traditional uses, and phytochemical contents of 96 species of *Alocasia.* The numerous biological activities of *Alocasia* species were also presented, which include anti-cancer, antidiabetic and antihyperglycaemic, antioxidant, antidiarrhoea, antimicrobial and antifungal, antiparasitic (antiprotozoal and anthelminthic), antinociceptive and anti-inflammatory, brine shrimp lethality, hepatoprotective, anti-hemagglutinin, anti-constipation and diuretic, and radioprotective activities as well as acute toxicity studies. Research articles were acquired by the accessing three scientific databases comprising PubMed, Scopus, and Google Scholar. For this review, specific information was obtained using the general search term “*Alocasia*”, followed by the “plant species names” and “phytochemical” or “bioactivity” or “pharmacological activity”. The accepted authority of the plant species was referred from theplantlist.org. Scientific studies have revealed that the genus is mainly scattered throughout Asia. It has broad traditional benefits, which have been associated with various biological properties such as cytotoxic, antihyperglycaemic, antimicrobial, and anti-inflammatory. *Alocasia* species exhibit diverse biological activities that are very useful for medical treatment. The genus *Alocasia* was reported to be able to produce a strong and high-quality anti-cancer compound, namely alocasgenoside B, although information on this compound is currently limited. Therefore, it is strongly recommended to further explore the relevant use of natural compounds present in the genus *Alocasia*, particularly as an anti-cancer agent. With only a few *Alocasia* species that have been scientifically studied so far, more attention and effort is required to establish the link between traditional uses, active compounds, and pharmacological activities of various species of this genus.

## Introduction

Plants from the Araceae family are classified as monocotyledonous flowering plants since the flower comes out from the inflorescence *spadix*. The Araceae family consists of 107 genera with over 3,700 species distributed worldwide (Erlinawati, 2010). The largest genera in the family are the *Alocasia,* which currently comprises 113 species with 27 species are still awaiting descriptions (Nauheimer et al., 2012). Of the total 96 accepted names of *Alocasia* species (GBIF, 2019), 79 species are reported as native to tropical and subtropical Asia, which extend from the subtropical eastern Himalayas throughout India, China, Japan, and across the Malay Archipelago until Oceania (Nauheimer et al., 2012; Ngoc-Sâm et al., 2017; Das, 2018; Ma et al., 2020). The genus *Alocasia* features tropical plants with large, often showy leaves, which are generally referred to as Elephant’s ear (Ongpoy, 2015; Yuliana & Fatmawati, 2018). The genus *Alocasia* is closely related to the genus *Colocasia*, which normally cause confusions between the two genera (Boyce, 2008; Srivastava et al., 2012).

Some *Alocasia* species are houseplants with high commercial value while others are grown outdoors, such as *Alocasia cucullata* (Lour.) G. Don (Chinese taro), an Asian plant of ethnobotanical importance and *A. macrorrhizos* (Lour.) G. Don (Giant taro), a tropical ornamental plant cultivated for its tubers and leaves, which is also used as animal fodder (Nauheimer et al., 2012). Besides being an ornamental plant, the genus *Alocasia* is traditionally used to treat several diseases including diarrhoea, constipation, diabetes, and cancer. Various phytochemicals have been identified in the *Alocasia* species such as flavonoids and phenolic compounds, contributing to its traditional uses (Das, 2018). Given this, several *in-Vivo* and *in-Vitro* studies have been conducted on the *Alocasia* species, particularly on the antioxidant properties and anti-tumour and cytotoxic studies (Ongpoy, 2017).

Therefore, the objective of this review was to explore the distribution of genus *Alocasia* around the world as well as to summarise their traditional uses, photochemical contents, *in-Vitro* and *in-Vivo* studies, and toxicology studies for future applications of the plant in medicinal and pharmaceutical fields.

## Materials and Methods

**FIGURE 1:**
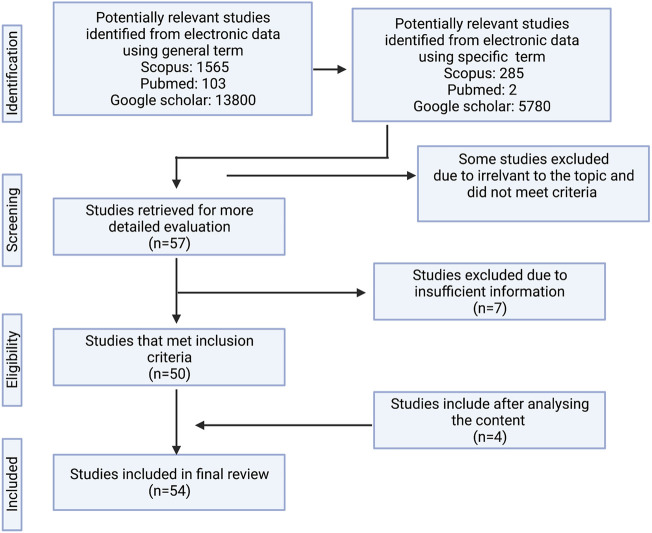
The flow diagram of the search method.

The review employed a partial systematic review protocol based on the PRISMA guidelines. The information was obtained through literature searches using three electronic databases: PubMed, Scopus, and Google Scholar. The selected references were not limited to any range of publication year. The primary search was based on the general term “*Alocasia*” while the following terms were used for secondary searches: “plant species names”, “phytochemicals”, “bioactivity”, and “pharmacological activities”. Certain references that were unable to be downloaded were excluded in the review. The remaining references were filtered to ensure that only articles covering the presence of *Alocasia* species, ethnobotanical surveys, traditional uses, chemical studies, *in-Vitro* and *in-Vivo* bioactivity studies were used in this review. Out of 13800 articles were found using general research term and in the end, we came up with 54 articles to undergo the review. In addition, the accepted authority of the plant species was referred from theplantlist.org. The layout of the searching methodology is presented in Figure 1 as adapted from Taher et al. (2020).

### Distribution

The genus *Alocasia* is native to Asia, Southeast Asia, and Australia. The distribution of *Alocasia* in tropical and subtropical forests with humid lowlands varies between regions and countries. For example, *A. macrorrhizos* (L.) G. Don [syn. *A. indica* (Lour.)] Spach is widely distributed in Bangladesh, Sri Lanka, India, and many other countries. It is among the oldest and the most common herbal plants that are rich in various nutrient compositions with different herbal medicinal properties to treat many diseases (Karim et al., 2014). It is also called the *Mankachu*, Giant *Alocasia*, Metallic taro, Giant Elephant taro, and Ape (Srivastava et al., 2012; Karim et al., 2014).

In addition, eight species including *A. odora* (Lindl.) K. Koch and *A. hypnosa* J.T. Yin, Y.H. Wang and Z.K. Xu has been reported and mostly found in the southeast region of China. *A. lihengiae*, a newly discovered species was found at the Jinuo mountains in southern Yunnan (Fang et al., 2020) while *A. yunqiana* was found at the Tongbiguan Nature Reserve in western Yunnan (Ma et al., 2020). *A. robusta* M. Hotta and *A. reversa* N.E. Br were found in the forest and on limestone rocks in Sarawak, Malaysia, respectively. Meanwhile, *A. sarawakensis* M. Hotta was found in Sabah, Malaysia, and *A. longiloba* Miq. syn. *A. denudata* was found in the understory of rainforest in Singapore (Nauheimer et al., 2012). Table 1 shows the distribution of *Alocasia* species around the world.

**Table 1:** Distribution of Alocasia species worldwide.

Species	Location	References
A. acuminata Schott	Nepal to China (S. Yunnan) and Indo-china, Thailand	Boyce (2008), GBIF, (2019)
A. aequiloba N. E. Br	Northern & Eastern New Guinea to Bismarck Archipelago	(Hay and Wise, 1991); GBIF, (2019)
A. alba Schott syn. A. crassifolia Engl	Southeast Sumatera to lesser Sunda islands	(Hay and Wise, 1991); GBIF, (2019)
A. × amazonica	Southeast Asia	GBIF, (2019)
A. arifolia Hallier f	Sumatera	GBIF, (2019)
A. atropurpurea Engl	Nansei-shoto (Okinawa, Iriomote), Philippines (N. Luzon)	GBIF, (2019)
A. augustiana L. Linden & Rodigas	Papua New Guinea	GBIF, (2019)
A. azlanii K. M. Wong & P. C. Boyce	Borneo (Brunei)	(Wong and Boyce, 2016); GBIF, (2019)
A. baginda Kurniawan & P. C. Boyce	Borneo (Kalimantan)	(Kurniawan and Boyce, 2011)
		GBIF, (2019)
A. balgooyi A. HAY	Sulawesi	GBIF, (2019)
A. beccarii Engl	Northwest Borneo	GBIF, (2019)
A. boa A. Hay	Papua New Guinea	(Hay and Wise, 1991); GBIF, (2019)
A. boyceana A. Hay	Philippines	GBIF, (2019)
A. brancifolia (Schott) A. Hay	Maluku, Papua New Guinea	(Hay and Wise, 1991); GBIF, (2019)
A. brisbanensis (F. M. Bailey) Domin	Northern and eastern Queensland to eastern South Wales	(Hay and Wise, 1991); GBIF, (2019)
A. cadieri Chantrier	Vietnam	GBIF, (2019)
A. celebica Engl. Ex Koord	Sulawesi (Minahassa Peninsula)	GBIF, (2019)
A. chaii P. C. Boyce	Borneo (Sarawak)	(Boyce, 2007); GBIF, (2019)
A. clypeolata A. Hay	Mindanao, Philippines	GBIF, (2019)
A. cucullata (Lour.) G. Don	Sri Lanka, Himalayas to South China and Indo-china	Wang et al., (2005); Xiao et al., (2014)
A. culionensis Engl	Philippines	GBIF, (2019)
A. cuprea K. Koch	Borneo (Sabah)	GBIF, (2019)
A. decipiens Schott	India to Myanmar, Andaman and Nicobar Islands	GBIF, (2019)
A. decumbens Buchet	Northern Vietnam	GBIF, (2019)
A. devansayana (L. Linden & Rodigas) Engl	Papua New Guinea	GBIF, (2019)
A. evrardii Gagnep. Ex V. D. Ngyuyen	Cambodia to Central Vietnam	GBIF, (2019)
A. fallax Schott	Eastern Himalaya to Bangladesh	GBIF, (2019)
A. farisii Zulhazman, Norziel. & P. C. Boyce	Peninsular Malaysia	Hamzah et al., (2017)
A. flabellifera A. Hay	Papua New Guinea	Hay and Wise, (1991); GBIF, (2019)
A. flemingiana Yuzammi & a. Hay	Western & Central Jawa	GBIF, (2019)
A. fornicata (Kunth) Schott	Northeast and southeast India to Indo-china, Sri Lanka	GBIF, (2019)
A. gageana Engl. *K. Krause	Northern Myanmar (Kachin Hills)	GBIF, (2019)
A. grata Prain ex Engl. & Krause	Southeast Myanmar	GBIF, (2019)
A. hainanica N. E. Br	Hainan to northern Vietnam	Wang et al., (2005)
A. hararganjensis H. Ara & M. A. Hassan	Bangladesh	Ara and Hassan, (2018); GBIF, (2019)
A. heterophylla (Presl) Merr	Philippines	Hay and Wise, (1991);GBIF, (2019)
A. hollrungii Engl	Noutheast Papua New Guinea to Bismarck archipelago	Hay and Wise, (1991);GBIF, (2019)
A. hypoleuca P. C. Boyce	Southeast Thailand	(Boyce, 2008);GBIF, (2019)
A. hypnosa	Thailand, China (Yunnan)	Wang et al., (2005); Boyce, (2008)
A. indica (Lour.) Spach	Indian subcontinent to Indo-china, Jawa	GBIF, (2019)
A. infernalis P. C. Boyce	Borneo (Sarawak)	(Boyce, 2007);GBIF (2019)
A. inornata Hallier f	Peninsula Malaysia to Sumatera	GBIF, (2019)
A. jiewhoei V. D. Nguyen	Cambodia	GBIF, (2019)
A. kerinciensis A. Hay	Sumatra	GBIF, (2019)
A. lancifolia Engl	Papua New Guinea	Hay and Wise, (1991); GBIF, (2019)
A. lauterbachiana (Engl.) A. Hay	Papua New Guinea to Bismarck Archipelago	Hay and Wise, (1991); GBIF, (2019)
A. lecomtei Engl	Vietnam	GBIF, (2019)
A. longiloba Miq	China (S. Yunnan, Guangdong) to western and Central Malaysia	Wang et al., (2005); Boyce, (2008)
*A. macrorrhizos* (L.) G. Don syn. *A. macrorrhiza*	Native to India, malaya, Thailand, vietnam	(Wang et al., 2005; Boyce, (2008)
*A. maquilingensis* Merr	Philippines	Hay and Yuzammi, (1998)
*A. megawatiae* Yuzammi & A. Hay	Sulawesi	GBIF, (2019)
*A. melo* A. Hay, P.C. Boyce & K. M. Wong	Borneo (Sabah, Malaysia)	GBIF, (2019)
*A. micholitziana* Sander	Luzon, Philippines	GBIF, (2019)
*A. miniuscula* A. Hay	Borneo (Sarawak)	GBIF, (2019)
*A. montana* (Roxb.) Schott	Eastern India	GBIF, (2019)
*A. monticola* A. Hay	Papua New Guinea	(Hay and Wise, 1991); GBIF, (2019)
*A. navicularis* (K. Koch & C. D. Bouche) K. Koch & C. D. Bouche	Nepal to China and Indo-china	(Boyce, 2008);GBIF, (2019)
*A. nebula* A. Hay	Borneo (Sarawak, Malaysia)	Hay, (2000)
*A. nicolsonii* A. Hay	Papua New Guinea	(Hay and Wise, 1991; Hay and Yuzammi, (1998)
*A. nycteris* Medecilo, G. C. Yao & Madulid	Philippines	GBIF, (2019)
*A. odora* (G. Lodd.) Spach	Eastern India to southeast Japan and Borneo	(Hay and Wise, 1991; Boyce, (2008)
*A. × okinawensis* Tawada	Nansei-shoto (Ryukyu islands), Japan	GBIF, (2019)
*A. pangeran* A. Hay	Borneo, Sabah	GBIF, (2019)
*A. peltata* M. Hotta	Borneo	GBIF, (2019)
*A. perakensis* Hemsl	Southeast Peninsular Thailand to Peninsular Malaysia	(Boyce, 2008); GBIF, (2019)
*A. portei* Schott	Luzon, Philippines	Hay and Wise, (1991)
*A. princeps* W. Bull syn. *A. porphyroneura* Hallier f	Borneo	GBIF, (2019)
*A. principiculus* A. Hay	Northern and eastern Borneo	GBIF, (2019)
*A. puber* (Hassk.) Schott	Peninsular Malaysia, western & Central Jawa	(Hay and Wise, 1991); GBIF, (2019)
*A. puteri* A. Hay	Borneo (Sabah)	GBIF, (2019)
*A. pyrospatha* A. Hay	Papua New Guinea	(Hay and Wise, 1991); GBIF, (2019)
*A. ramosii* A. Hay	Philippines	GBIF, (2019)
*A. reginula* A. Hay	Borneo	GBIF, (2019)
*A. reversa* N. E. Br	Borneo (Sarawak)	GBIF, (2019)
*A. ridleyi* A. Hay	Borneo (Sarawak)	GBIF, (2019)
*A. rivularis* Luu, Nguyen-Phi & T. T. Van	Vietnam	GBIF, (2019)
*A. robusta* M. Hotta	Borneo	GBIF, (2019)
*A. salarkhanii* H. Ara & M. A. Hassan	Bangladesh	GBIF, (2019)
*A. sanderiana* W. Bull	Mindanao, Philippines	GBIF, (2019)
*A. sarawakensis* M. Hotta	Borneo	Hay and Yuzammi, (1998)
*A. scabriuscula* N. E. Br	Borneo	(Hay and Wise, 1991); GBIF, (2019)
*A. scalprum* A. Hay	Samar island, Philipines	GBIF, (2019)
*A. simonsiana* a. hay	Papua New Guinea	GBIF, (2019)
*A. sinuata* N. E. Br	Philippines	GBIF, (2019)
*A. suhirmaniana* Yuzammi & A. Hay	Southeast Sulawesi	Hay and Yuzammi, (1998)
*A. venusta* A. Hay	Borneo (North Sarawak)	GBIF, (2019)
*A. vietnamensis* V. D. Nguyen & de Kok	Central Vietnam	GBIF, (2019)
*Alocsia wentii* Engl. & K. Krause	New Guinea (Mt. Hellwig, Star mountains)	(Hay and Wise, 1991); GBIF, (2019)
*A. wongii* A. Hay	Borneo (Sabah)	GBIF, (2019)
*A. Zebrina* schott ex Van Houtte syn. *A. liervalii* Herincq or *A. wenzelii* Merr	Philippines	GBIF, (2019)

Several notable studies have also been conducted to re-evaluate the genus *Alocasia* especially in Australasia (Hay & Wise, 1991), the Philippines (Hay, 1999), Borneo (Hay, 2000; Kurniawan & Boyce, 2011), Thailand (Boyce, 2008), and Peninsular Malaysia (Hamzah et al., 2017). While the number of identified *Alocasia* species have increased steadily over the last 2 decades (Ma et al., 2020), it is expected that more species are to be discovered in the future, allowing new molecules from the species to be studied (Ongpoy Jr, 2017).

### Botany

*Alocasia* species are predominantly humid lowland tropical plants and are terrestrial diminutive geophytes (Hay & Wise, 1991; Ongpoy Jr, 2017; Das, 2018; Ma et al., 2020). Some species are able to grow on trees while very few can propagate underwater (Ma et al., 2020). In addition, only a few species are known to grow at an altitude above 1000 m or in light gaps, clearings, or secondary vegetation (Das, 2018). Certain species such as *A. perakensis* Hemsl and *A. kerinciensi* A. Hay usually grow in mountainous areas and are found above an altitude of approximately 1200 m above sea level, but not exceeding an altitude of approximately 2000 m above sea level. Some of these species live on the rocks, including *A. longiloba* “watsoniana”, *A. longiloba* ”*lowii*”*, A. principiculus* A. Hay*, A. puteri* A. Hay*, A. princeps* W. Bull*, A. ridleyi* A. Hay*, A. venusta* A.Hay, and *A. reversa* N.E. Br.

Furthermore, *A. melo* A. Hay, P.C. Boyce and K.M. Wong only lives in ultramafic regions (frozen plutonic and metamorphic rocks). *A. reversa*, *A. venusta*, *A. ridleyi*, *A. princeps*, and *A. principilus* A. Hay were confined to limestone areas. *A. miniscula* A. Hay is known to be found only in peat swamp forests*. A. cuprea* K. Koch is found in sandstone, limestone, and ultramafic areas while *A. princeps* can live on various types of media, limestone, sandstone, and shale. Meanwhile, *A. robusta*, *A. sarawakensis*, *A. alba* Schott, *A. puber* (Hassk.) Schott, *A. scabriuscula* N.E. Br, *A. inornata* Hallier f., and *A. longiloba* are found in the forest (Hay & Wise, 1991).

As robust vegetative plant herbs, the growth of *Alocasia* species ranges from small herbaceous to massive plants with thick stems and large leaves (Das, 2018). The complex floral structures of *Alocasia* species are characterised by the plant’s leaves that are occasionally subtended by a cataphyll with several terminal crowns. The long petioles are either aspirate or glandular while the leave blade is peltate at juvenile which changes to sagittate upon maturity. In addition to the flowers that are unisex with no perigone, the plant is characterised by its unique properties, which include the production of clear or slightly milky sap, the formation of the synandria from the flower juice, and the ripe to the orange-red colour of the fruit (Hamzah et al., 2017). The seeds of the plants are dispersed mainly through birds while the drosophilid flies (genus *Colocasiomyia*) use spadices as the breeding site to pollinate the plants (Das, 2018).

### Traditional Uses

A strong bond between human beings and plants have existed since ancient time as the use of plants in treating numerous diseases have always been a central part in human’s life (Basu et al., 2014). This relationship remains today with 80% of the population in developing countries still utilises traditional plant-based medicines instead of pharmaceutical drugs due to their efficacy, easy accessibility, affordability, and lesser toxic effects (Srivastava et al., 2012; Nahdi & Kurniawan, 2019). *Alocasia* species is not only known as a vegetable but it is also used as ornamental and medicinal plants (Ongpoy Jr, 2017). *A. indica* Linn is traditionally used to treat snake and tiger bites, rheumatoid arthritis, and hives (Houghton & Osibogun, 1993; Bakar et al., 2009) while *A. macrorrhizos* are traditionally used for the treatment of diabetes, pus in the ears, jaundice, and constipation (Rahman et al., 2012).

The healing practices of *Alocasia* species vary based on the knowledge and traditions of the respective cultures, ethnicity, or regions (Basu et al., 2014; Nahdi & Kurniawan, 2019). *A. macrorrhiza* (Linn.) Schott is widely used in many countries. Among them, it is used traditionally for cough and toothache in Malaysia (Ongpoy Jr, 2015; 2017), as an analgesic medication to alleviate pain in the stomach, head, and rheumatoid arthritis in India, Sulawesi, and Bangladesh, respectively (Al Hassan et al., 2014; Yuliana & Fatmawati, 2018), as well as to treat inflammation, eczema, and abscess in Vietnam (Yuliana & Fatmawati, 2018). *A. longiloba* syn. *A. denudata* Engl. or locally known as “keladi candik” in Kelantan, Malaysia is traditionally used to accelerate wound healing and as an anti-inflammatory remedy (Abdulhafiz et al., 2020). In addition, *A. brisbanensis* is used traditionally by the Yaegl Aboriginal community of New South Wales for burns and boils, cuts, sores, and open wounds (Packer et al., 2015). In China, the Zhuang ethnic used the tuber of *A. cucullata* as a detoxification drink, to reduce swelling, and to ease pain (Wei et al., 2015) while the Wonokerto people in Yogyakarta used *A. plumbea* K. Koch ex Van Houtte as a traditional remedy to treat thypus (Nahdi & Kurniawan, 2019).

Furthermore, the plant extract and distinct parts of the *Alocasia* plant such as the leaf, stem, tuber, or rhizome are used to treat different types of diseases. Stem juice of *A. macrorrhiza* is applied to prevent oedema, pain, and bleeding from cuts and wounds while the leaves are used to prevent iron deficiency and to enhance eyesight (Rahman et al., 2012). In India, the rhizome paste is used by the Konda Reddis and Savaras tribes to treat wounds and to kill worms in domestic animals. Rhizome pastes are also used by the Kanda and Nuke Dora’s tribes to cure heel cracks and wounds (Haque et al., 2014). Moreover, the leaf stalk of *A. macrorrhiza* is boiled in water and drank or is eaten raw by the traditional people of Cikondang village in West Java to treat coughs (Kodir et al., 2017). The bulb of *A. macrorrhiza* is also useful as a remedy to reduce constipation by people of the Chawan district in Thailand (Neamsuvan et al., 2016). *A. indica* (Roxb.) Schott is used by the community in Khulna District, Bangladesh to treat tiger bites, rheumatoid arthritis, and itching (Rahmatullah et al., 2010), while the leaves and roots are effective to treat snake bites (Houghton & Osibogun, 1993).

Interestingly, besides using to treat sore eyes, the people of Pulo Adat Village have traditionally used the leaves of *A. plumbea* K. Koch ex Van Houtte as protection from ghost disturbances in ritual processes (Widyastuti et al., 2019)**.** With a few exceptions on the plants’ folkloric uses, intensive studies on the *Alocasia* species are crucial for novel drug discovery and its medicinal application since many of the species remain unexplored. Table 2 shows the difference in traditional uses of *Alocasia* species in different countries.

**TABLE 2:** Traditional uses of Alocasia species in different countries.

Species	Uses	Plant organs	Form of uses/Route of administration	References
*A. brisbanensis* Domin	Burns, cuts, ulcers, and open wounds	Leaves, stems	Not available	
*A. cucullata* (Lour.) G.Don	Detoxify viper bites	Roots	Applied externally	Houghton and Osibogun (1993)
	Detoxify snakebites	Rhizomes	Use as decoction form and applied as external baths and poultices	Otero et al. (2000)
*A. indica* Schott	Rubefacient, external stimulant and for fever	Rhizomes	Not available	
	Diabetes	Rhizomes	Decoction	Srivastava et al. (2012)
*A. longiloba* Miq	Coughs and fever	Petioles	Boiled, then drunk or eaten or make a juice	Abdulhafiz et al., (2020)
				Widyastuti et al. (2019)
	Relieve pain due to inflammation and heal wounds	Petioles	Paste and externally applied to the wounded areas	
	Treat furuncles	Rhizomes	Used as poultice	Neamsuvan et al. (2016)
	Reduces pain due to neck swelling, constipation and hemorrhoids	Bulbs	Not available	
	Used for gout, rheumatism and constipation	Bulbs	Mashed the tuber and the juice is drank	Srivastava et al. (2012)
*A. macrorrhizos* (L). G. Don	Digestive laxative, diuretic, astringent and traditionally used for the treatment of rheumatic arthritis	Leaves	Juices	Kaur et al., (2005); Srivastava et al., (2012)
	To treat colic and constipation	Leaves and stems	Boiled together and serve with ghee for 3 consecutive days	Das (2018)
	Rubefacient	Leaves and roots	Chopped up together and applied externally	
	To treat generalized edema, hemorrhoids and habitual constipation	Root and stem (tuber)	Conjee made of the root-stock or dried stem (tuber) boiled with rice flour	
	Gout and rheumatism	Tuber	Applied locally to painful area after heating the tubers	
	Rheumatic pain	Tuber	Dried and powdered tuber is taken orally with milk and sugar following boiling on a daily basis	
	Constipations and piles	Tuber	The tuber is in powdered form and taken orally	Neamsuvan et al. (2016)
	Mouth ulcer	Root	Mixed the root-stock with honey and apply locally to the affected area	Srivastava et al. (2012)
	Toothache	Petioles	Applied locally	Srivastava et al. (2012)
	Cough and otorrhea	Petioles	Make a juice and dropped into the ears of children	Srivastava et al., (2012); Das (2018)
	Laxative	Stem	-	Das (2018)
	Treat scorpion stings	Stem	-	Das (2018)
*A. plumbea* K. Koch ex. Van Houtte	Minor eye pain	Leaves	Drop water from the leaf into eyes externally	Widyastuti et al. (2019)
*A. fornicata* (Roxb.) Schott	Treat wounds, cure heel cracks and kill worms in domestic animals	Rhizomes	Paste	Haque et al., (2014); Das (2018)
	Prevent edema, pain and bleeding from cuts and wounds	Stem	Juice	Karim et al. (2014)
	Treat pus in ears, jaundice and constipation	Whole plant	-	
	Painful joints	Roots and leaves	Chopped and applied directly	
	Rubefacient, external stimulant and for fevers	Rhizomes		

### Phytochemistry

Reactive Oxygen Species (ROS) or free radicals are found in the human biological system and can damage various molecules such as DNA and inhibit cell function, leading to the development of disease (Abdulhafiz et al., 2020). The bioactive compounds or secondary metabolites found in plants, fruits, and vegetables are able to scavenge these ROS in the human body, making them beneficial and effective in treating various chronic diseases or disorders such as cardiovascular disease, cancer, obesity, diabetes, hyperuricemia, gout, and inflammatory diseases (de la Rosa et al., 2018; Abdulhafiz et al., 2020). A broad range of biochemical compounds can be obtained from natural sources through an extraction method in which specific dosage forms can be prepared for pharmaceutical purposes (Basu et al., 2014).

Phenolic compounds are the most abundant biomolecules in plants and are responsible for the plant’s defence mechanisms (de la Rosa et al., 2018; Abdulhafiz et al., 2020). The compounds possess complex structures, which give colour, flavour, structural support, and protection to the plants against microorganisms (Karim et al., 2014; de la Rosa et al., 2018). In addition, they exhibit various biological properties such as antioxidant, anti-inflammatory, and anti-cancer which serve as a health remedy for human against several chronic diseases (de la Rosa et al., 2018; Abdulhafiz et al., 2020). At various stages, the compounds control many cellular processes, including enzyme inhibition, gene expression modulation, and protein phosphorylation (de la Rosa et al., 2018).

**FIGURE 2:**
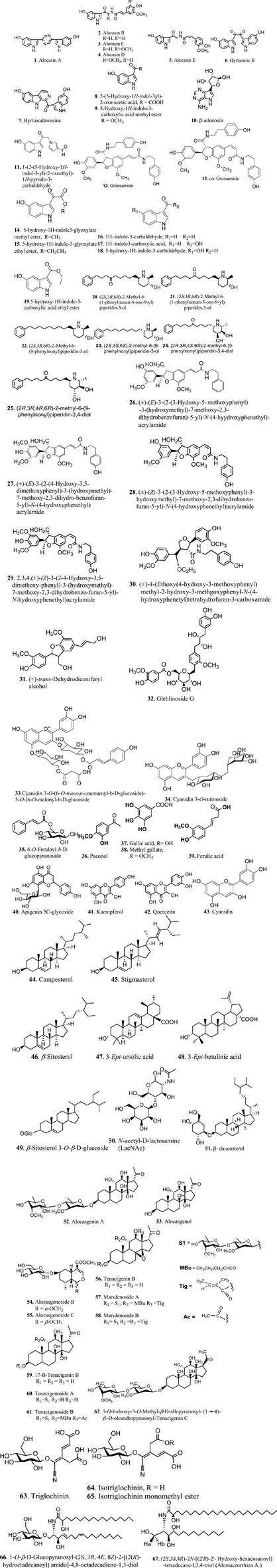
Chemical structure of phytochemicals reported from Alocasia species.

Among the bioactive compounds that have been identified in *Alocasia* extracts are alkaloids, flavonoids, and phenolic compounds which have high medicinal values including antioxidant, anti-cancer, anti-inflammatory, antimicrobial, antihyperglycaemic, antidiarrhoea, and antidiabetic (Karim et al., 2014; Yuliana & Fatmawati, 2018; Abdulhafiz et al., 2020). In addition, different parts or extracts of the *Alocasia* plants contain different types and amounts of bioactive metabolites. For instance, the rhizome extract of *A. macrorrhiza* is rich in flavonoid contents (Yuliana & Fatmawati, 2018) while the stem of *A. indica* contains a high concentration of phenolic contents compared to the leaves sample (Karim et al., 2014). List of compounds that have been reported in the references are listed in Figure 2. Based on the previous study, it was reported that from *Alocasia* species was mainly contains alkaloids (30 compounds), sterols (12 compounds), triterpenoids and flavonoids (3 compounds each) and iridoids 2) compounds, sphingolipid, and ceramide (1 compound each).

In addition, summary of the compounds and parts used in each species of some *Alocasia* is tabulated in Table 3.

**TABLE 3:** Summary phytochemicals and part used of some *Alocasia* species.

Phytochemicals	Spesies	Compounds	Part of the plant	References
Alkaloids	*A. macrorrhiza* (L.) Schott	Alocasin A **(1),** alocasin B **(2),** alocasin C **(3),** alocasin D **(4),** alocasin E **(5),** hytiosin B **(6),** hyrtiosulawesine **(7)**	Ethanolic extract of the rhizomes	Zhu et al. (2012)
	*A. macrorrhiza* (L.) Schott	Alocasin B **(2)**, Hyrtiosin B **(6),** 2-(5-Hydroxy-1*H*-indol-3yl)-2-oxo-acetic acid **(8)**, 5-Hydroxy-1*H*-indole-3-carboxylic acid methyl ester **(9)**	Methanolic extract of the rhizomes	Elsbaey et al., (2017)
	*A. cucullata* Schott	*β*-adenosine**,** 1*H*-indole-3-carbaldehyde (**10**)	Ethanolic extract of the tubers	Xiao et al. (2014)
Indole alkaloidal	*A. macrorrhiza* (L.) Schott	1-(2-(5-Hydroxy-1*H*-indol-3-yl)-2-oxoethyl)-1*H*-pyrrole-3- carbaldehyde **(11)**	Ethanolic extract of the rhizomes	Huang et al. (2017b)
	*A. macrorrhiza* (L.) Schott	Grossamide (**12**), cis-grossamide (**13**), 5-hydroxy-1H-indole- 3-glyoxylate methyl ester (**14**), 5-hydroxy-1H-indole-3-glyoxylate ethyl ester (**15**), 1H–indole-3-carbaldehyde (**16**), 1H–indole- 3-carboxylic acid (17), 5-hydroxy-1H-indole-3-carbaldehyde (**18**), and 5-hydroxy-1H-indole-3-carboxylic acid ethyl ester (**19**)	Ethanolic extract of the rhizomes	Huang et al. (2017a)
Piperidine alkaloids	*A. macrorrhiza* (L.) Schott	(2*S*,3*R*,6*R*)-2-methyl-6-(1-phenylnonan-4-one-9-yl) piperidin-3-ol **(20),** (2S,3R,6R)-2-methyl-6-(1-phenylnonan-5-one-9-yl) piperidin-3-ol **(21),** (2S,3R,6R)-2-methyl-6-(9- phenylnonyl)piperidin-3-ol **(22),** (2*S*,3*S*,6*S*)-2-methyl-6-(9-phenylnonyl)piperidin-3-ol**(23),** (2*R*,3*R*,4*S*,6*S*)-2-methyl-6-(9-phenylnonyl)piperidine-3,4- diol **(24),** (2*R*,3*R*,4*R*,6*R*)-2-methyl-6-(9-phenylnonyl)piperidine-3,4- diol **(25)**	Ethanolic extract of the rhizomes	Huang et al. (2017b)
Lignanamides	*A.macrorrhiza* (L.) Schott	(±)-(*E*)-3-(2-(3-Hydroxy-5-methoxyphenyl)-3-(hydroxymethyl)- 7-methoxy-2,3-dihydrobenzofuran-5-yl)-N-(4-hydroxyphenethyl)acryl- amide **(26)**	Ethanolic extract of the rhizomes	Huang et al. (2017a)
		(±)-(*E*)-3-(2-(4-Hydroxy-3,5-dimethoxyphenyl)-3-(hydroxymeth-yl)-7-methoxy-2,3-dihydrobenzofuran-5-yl)-N-(4-hydroxyphenethyl)acrylamide **(27)**		
		(±)-(*Z*)-3-(2-(3-Hydroxy-5-methoxyphenyl)-3-(hydroxymethyl)- 7-methoxy-2,3-dihydrobenzofuran-5-yl)-N-(4-hydroxyphenethyl)acryl- amide **(28)**		
		(±)-(*Z*)-3-(2-(4-Hydroxy-3,5-dimethoxyphenyl)-3-(hydroxymeth- yl)-7-methoxy-2,3-dihydrobenzofuran-5-yl)-N-(4- hydroxyphenethyl)acrylamide **(29)**		
		(±)-4-(Ethoxy(4-hydroxy-3-methoxyphenyl)methyl)-2-(4-hy- droxy-3-methoxyphenyl)-N-(4-hydroxyphenethyl)tetrahydrofuran-3- carboxamide **(30)**		
Lignans	*A. cucullata* Schott	(+)-trans-dehydrodiconiferyl alcohol (**31**), glehlinoside (**32**)	Ethanolic extract of the tubers	Xiao et al. (2014)
Anthocyanins	*A. cucullata* Schott	Cyanidin 3-*O*-(6-*O*-trans-*p*-coumaryl-β-D-glucoside)-5-*O*-(6-*O*-malonyl- β—D-glucoside) (**33**)	Ethanolic extract of the tubers	Xiao et al. (2014)
	*A. cuprea* (C. Koch & Bouche) C. Koch	Cyanidin 3-rutinoside (**34**)	Leaves	Williams et al. (1981)
	*A. macrorrhiza* (L.) G. Don var. *rubra* (Hassk.) Furtado	Cyanidin 3-rutinoside (**34**)	Petiole	Williams et al. (1981)
	*A. porteii* Schott	Cyanidin 3-rutinoside (**34**)	Leaves	Williams et al. (1981)
	*A. thibantiana* Mast. Cv Silver King syn *A. suhirmaniana* Yuzammi & A. Hay	Cyanidin 3-rutinoside (**34**)	Leaves	Williams et al. (1981)
	*A. lauterbachiana* (Engl.)	Cyanidin 3-rutinoside(**34**)	Leaves and stems	Williams et al. (1981)
Phenylpropanoids	*A. cucullata* Schott	6-*O*-feruloyl- *β*—D-glucopyranoside (**35**)	Ethanolic extract of the tubers	Xiao et al. (2014)
Phenolic acids	*A. cucullata* Schott	Paeonol (**36**)**,** gallic acid (**37**)**,** methyl gallate(**38**)**,** ferulic acid (**39**)	Ethanolic extract of the tubers	Xiao et al. (2014)
Flavonoids	*A. alba* Schott	Apigenin 5*C*-glycoside **(40)**, kaempferol **(41)**	Methanolic extract of the leaves	Williams et al. (1981)
	*A. cuprea* C.Koch & Bouche	Apigenin 5*C*-glycoside **(40)**, kaempferol **(41),** quercetin **(42)**, cyanidin **(43)**	Methanolic extract of the leaves	Williams et al. (1981)
	*A cucullata* (Lour.) G. Don	Apigenin 5*C*-glycoside **(40)**	Methanolic extract of the leaves	Williams et al. (1981)
	*A. lauterbachiana* (Engler) A. Hay	Apigenin 5*C*-glycoside **(40)**, quercetin **(42)**, cyanidin **(43)**	Methanolic extract of the leaves	Williams et al. (1981)
	*A. macrorrhizos* var. *variegata* (K.Koch & C.D.Bouché) Furtado	Apigenin 5*C*-glycoside **(40)**, kaempferol **(41),** quercetin **(42)**, cyanidin **(43)**	Methanolic extract of the leaves	Williams et al. (1981)
	*A. macrorrhizos* var. rubra (Hassk.) Furtado	Apigenin 5*C*-glycoside **(40)**, quercetin **(42)**, cyanidin **(43)**	Methanolic extract of the leaves	Williams et al. (1981)
	*A. macrorrhizos* var. rubra (Hassk.) Furtado	Apigenin 5*C*-glycoside **(40)**, quercetin **(42)**, cyanidin **(43)**	Methanolic extract of the leaves	Williams et al. (1981)
	*A. odora* (Roxb) C. Koch	Apigenin 5*C*-glycoside **(40)**, cyanidin **(43)**	Methanolic extract of the leaves	Williams et al. (1981)
	*A. porteii* Schott	Apigenin 5*C*-glycoside **(40)**	Methanolic extract of the leaves	Williams et al. (1981)
	*A. thibantiana* Mast. Cv Silver King syn *A. suhirmaniana* Yuzammi & A. Hay	Apigenin 5*C*-glycoside **(40)**, kaempferol **(41),** quercetin **(42)**, cyanidin **(43)**	Methanolic extract of the leaves	Williams et al. (1981)
Sterols	*A. indica* Schott	Campesterol **(44)**, stigmasterol **(45)**, *β*-sitosterol **(46)**	Ethanol extract of the rhizomes	Basu et al. (2014)
	*A. denudata syn A. longiloba*	Campesterol (**44**) Stigmasterol (**45**)**,** β-sitosterol (**46**)	Ethanolic extract of the stems	Mohd Yusoff et al. (2020)
	*A. macrorrhiza* (L.) Schott	*β*-Sitosterol **(46)**, 3-*Epi*-ursolic acid **(47),** 3-*Epi*-Betulinic acid **(48),** *β*-sitosterol 3-*O-β*-D-glucoside **(49)**	Methanol extract of the rhizomes	(Elsbaey et al. (2017)
Lectins	*A. cucullata* (Lour.) G. Don	*N*-Acetyl-D-lactosamine (LacNAc) **(50)**	Tubers	(Kaur et al., 2005; Xiao et al., 2014)
Saponins	*A. cucullata* (Lour.) G. Don	*β*-daucosterol (**51**)	Ethanolic extract of the tubers	Xiao et al. (2014)
	*A. cucullata* (Lour.) G. Don	Alocasgenin A **(52)**, alocasgenol **(53)**, alocasgenoside B **(54)**, alocasgenoside C **(55)** tenacigenin B **(56)**, marsdenoside A **(57)**, marsdenoside B **(58)**, 17- *β*-Tenacigenin B **(59)**, 3-*O*-6-deoxy-3-*O*-methyl- *β*-D-allopyranosyl- (1–4)—β -D-oleandropyranosyl-tenacigenin C (**60**), tenacigenoside A **(61),** dan tenacigenoside B **(562)**	Ethanolic extract of the tubers	Peng et al. (2016)
Cyanogenic glycosides	*A. macrorrhiza* Schott	Triglochinin (**63**) and isotriglochinin (**64**)	Leaves	Nahrstedt (1975)
Sphingolipids	*A. macrorrhiza* (L.) Schott	1-*O-β-D*- glucopyranosyl-(2*S*,3*R*,4*E*,8*Z*)-2-[(2(*R*)–hydroctadecanoyl)amido]-4,8-octadecadiene-1,3-diol (**65**)	Methanolic extract of the rhizomes	Elsbaey et al. (2017)
Ceramide	*A. macrorrhiza* (L.) Schott	(2*S*,3*S*,4*R*)-2*N*-[(2′*R*)-2′- Hydroxy-hexacosanoyl]-tetradecane-l,3,4-triol (alomacrorrhiza A) **(66)**	Ethanolic extract of the roots	Tien et al. (2004)

### Biological Activity

In this review, the main biological activities of *Alocasia* species discussed include anti-cancer, antidiabetic and antihyperglycaemic, antioxidant, antidiarrhoea, antimicrobial and antifungal, antiparasitic (antiprotozoal and anthelminthic), antinociceptive and anti-inflammatory, brine shrimp lethality, hepatoprotective, anti-hemagglutinin, anti-constipation and diuretic, and radioprotective activities.

### Anti-Cancer Activity

Cancer is the leading cause of death worldwide, accounting for 7.4 million deaths, or approximately 13% of all deaths, in 2004 alone. The most common causes of cancer death annually (death/year) were lung cancer (1.3 million), followed by stomach cancer (803,000), colorectal cancer (639,000), liver cancer (610,000), and breast cancer (519,000). In fact, cancer deaths worldwide are projected to rise continuously with an estimated 11.5 million deaths by 2030 (World Health Organization, 2009). Cancer is caused by the alteration of gene expression in cells, leading to an abnormality in cell growth. Plants have been recorded to be a source of anticancer agents. There are drugs that available in clinical setting such as paclitaxel, a taxan derivative which was derived from *Taxus bervifolia* Nutt (Western yew) (Fridlender et al., 2015) and viscumin, a lectin derivative, from *Viscum album* L. ( Olsnes et al., 1982; Buyel, 2017 ).

A lectin derivative, N-acetyl-D-lactosamine (**50**) was the earliest cytotoxic compound from *A. cucullata (Lour.)* G. Don tuber which was active against various human cancer cell lines at concentrations ranging between 10 and 100 μg/ml. It was found that the optimum *in-Vitro* antiproliferative activity towards human cancer cell lines with lectin was at a concentration of 100 μg/ml. In addition, significant growth inhibition of 50% and 46% was observed in SiHa (cervix) and PC-3 (prostate) cell lines, respectively. However, the central nervous system (SNB-78) and breast cancer cell line (A-549) recorded only 16% and 23% of growth inhibition, respectively. A number of possible mechanisms of the antiproliferative effect exhibited by compound **50** were highlighted including the ability of compound **50** to stimulate the immune response, enhancing the activity of lymphocytes in tumour-bearing mice, inhibiting the synthesis of protein in several malignant cell lines, and interact specifically with carbohydrate on the surface of tumour cell which distinguished malignant cells from the normal cells.

Cytotoxic activity of *A. macrorrhizos* tuber on human throat cancer (Hep-2), human hepatocarcinoma (Hep-G2), and human nasopharyngeal carcinoma epithelial (CNE) reported a mild antiproliferative activity against Hep-2 and Hep-G2 which was demonstrated by compounds alocasin A (**1**), alocasin B **2**) alocasin C (**3**), alocasin D (**4**), and alocasin E (**5**) , hyrtiosine B (*6*), whereas hyrtiosulawesin (**7**) and showed gentle antiproliferative activity against CNE (Zhu et al., 2012). Furthermore, Lei et al. (2012) reported that ethanol extracts of *A. macrorrhiza* rhizome contained lipid contents such as (*Z*, Z)-9,12-octadecadienoic acid, hexadecanoic acid, 3-pentadecylphenol, (*Z*, *Z*, *Z*)-9,12,15-octadecatrienoic acid, and 3′-methoxybenzo [1′, 2′-b]-1,4-diazabicyclooctene which may contribute to the *in-Vitro* anti-cancer activity of the plant against gastric cancer cellsM-GC803. This study suggested that not only secondary metabolites exhibit anti-cancer properties but primary biochemical compounds also act as an anti-cancer agent.

The results from the *in-Vitro* study of *A. macrorrhiza* showed the inhibition of cell proliferation and apoptosis induction on human hepatocellular cells (SMMC-7721) at a concentration of 400 μg/ml. In addition, the *in-Vitro* study using MTT assay found that the aqueous extract of *A. macrorrhiza* exhibited a dose- and time-dependant inhibitory effect ranging between 100 and 500 μg/ml. The expression levels of the potent cell proliferation inhibitor (PPAR-γ), pro-apoptotic proteins (Bax), and active caspase-3 increased in a dose-dependent manner while the expression of Cyclin D1 (the protein required to accelerate cell cycle progression) and anti-apoptotic protein (Bcl-2) decreased in a dose-dependent manner (Fang et al., 2012). The *in-Vivo* anti-tumour activities of the same extract (concentration ranging between 0.2 and 0.8 g/kg/day) against murine hepatoma (H22) cells inoculated in mice recorded a decrease in the mean tumour weight in a dose-dependent manner. Moreover, the concentration at 0.8 g/kg/day of the water-soluble of *A. macrorrhiza* showed the lowest mean tumour weight values with the highest inhibitory rates without any untoward toxicity. The mechanism of anti-tumour activity of the extract could be associated with the inhibition of DNA synthesis, cell cycle (G0/G1) arrest stimulation, apoptosis induction through up-regulation of the mRNA and protein expressions (PPAR-γ, Rb, Baz, and caspase-3 genes), and the down-regulation expressions of Cyclin D1 and Bcl-2 genes (Fang et al., 2012).

Another cytotoxic species that had been reported is *Alocasia cucullate. A. cucullata* root was effective *in-Vivo* against breast tumour (4TI)-bearing mice at high dose (16 g/kg/day) compared to medium dose (8 g/kg/day) and low dose (4 g/kg/day). The high dose of aqueous *A. cucullata* extract also showed significant attenuation of the tumour growth, prolongation of the mice survival, and reduction in tumour weight and size. The study suggested that the *in-Vivo* attenuation of tumour growth using a high dose of *A. cucullata* extract could be due to the ability of the extract to increase the spleen size and enhance the anti-tumour human immune response of the mice, subsequently induced key cytokines *in-Vivo* such as IL-2, IFN-γ, and TNF-α to further stimulate various immune cells and immune activity (Peng et al., 2013).

In *in-Vitro* study, *A. cucullata* root water extract against various cultured mammalian cells comprising cervical cancer cell (HeLa), osteosarcoma cell (U-2OS), lung cancer cell (A549), and retina pigment epithelial cell (RPE). Despite that all concentrations of *A. cucullata* extract exhibited weak anti-cancer activity with a maximum concentration of 2 mg/ml, the concentration of the extract at 2 mg/ml was able to stimulate differentiation of human monocytic cell line, THP-1 to macrophage cells by 48%, thus increase the production of cytokines (IL-1β and TNF-α) in a dose-dependent manner. Nonetheless, the stimulation exhibited by the positive control was twice higher than the extract, which was 95% at the same concentration (Peng et al., 2013).

**FIGURE 3:**
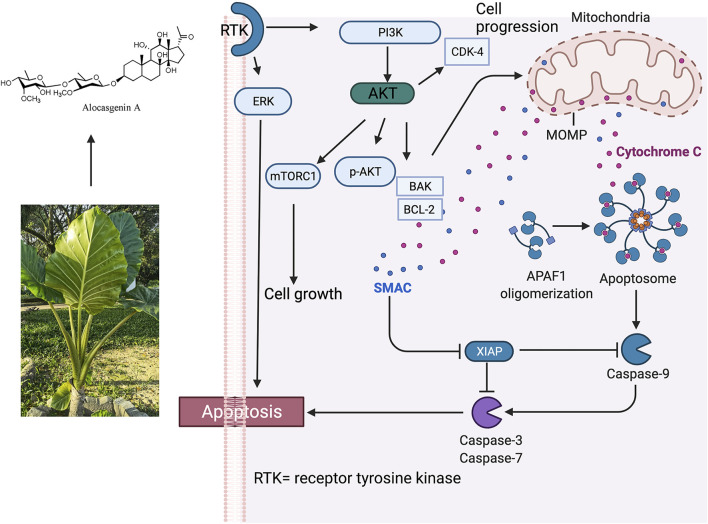
Proposed antitumor activity of *Alocasia via* intrinsic apoptosis. Phosphorylation of Bax by Akt or activated Akt disables its translocation to the mitochondrial membrane, then decreases permeabilization. p-AKT was significantly decreased in a dose-dependent manner, while the treatment did not change AKT expression. It was also found that the expression of Bcl-2 protein decreased and the expression of Bax protein increased which led to an increase of the Bax/Bcl-2 ratio. It was postulated that *Alocasia* works *via* following mechanism: 1) PI-3 K/Akt pathway of apoptosis; 2) ERK activity of apoptosis; 3) triggering of mitochondrial pathway; 4) connection by Bcl-2 and Bax; v.G0/G1 cell cycle arrest (Wei, et al., 2015). Alocasgenin A represents anticancer compound from *Alocasia*.

*A. cucullata* also active against gastric cancer (MGC-803), breast cancer (MDA-MB-435), myelogen leukaemia cancer (K-562), and liver carcinoma (Bel7402) with maximum concentration at 50 μg/ml after 48 h of treatment in a dose-dependent manner. The highest susceptibility to the EAC-B was displayed by MGC-803 with the IC_50_ value of 42.1 ug/mL. Moreover, the *in-Vivo* study showed that 1 g/kg and 5 g/kg of EAC-B exhibited 50–90% of anti-tumour activity, particularly through the reduction of the tumour growth and increased necrosis in MGC-803-injected mice. More importantly, the varying doses did not show any significant difference in the anti-tumour activity and it was suggested that the maximum dose at 5 g/kg possessed a low toxicity profile for the mice. The EAC-B treated MGC-803 cells may induce apoptosis by inhibition of the AKT and ERK pathways, following the involvement of Bcl-2, Bax, and cytochrome C release and apoptosis action by caspase 3/7 (Wei et al., 2015). The proposed antitumor activity of *Alocasia* is presented in Figure 3.

The active principles in *A. cucullata* tuber, **52, 53, 54, 55, 56**, and **60** showed *in-Vitro* cytotoxic activities against gastric cancer cell line (MGC-803) and colon cancer cell line (HT-29) and were suggested as active ingredients against gastrointestinal cancer. Compound **54** strongly inhibited the growth of MGC-803 and HT-29 with IC_50_ of 0.98 ± 0.3 μg/ml and 1.56 ± 0.8 μg/ml, respectively besides displaying inhibitory effects on tyrosine kinase. Meanwhile, compounds **52**, **53**, **55**, **56**, and **60** showed moderate cytotoxicity activity as the IC_50_ values of these compounds were higher than that of compound **54**. The mechanism of anti-tumour of compound **54** was suggested *via* the phosphoinositide 3-kinase/AKT (PI3K/AKT) pathway. Compound **55**, which is an isomer of compound **54**, exhibited a much lesser cytotoxic activity which may be related to their different configuration (Peng et al., 2016).

Hyrtiosin B **(6)** and 1-O-β-D-glucopyranosyl-(2S, 3R, 4E, 8Z)-2-{[2(R)-hydroctadecanoyl]amido}-4,8-octadecadiene-1,3-diol (**65**) from *A. macrorrhiza* demonstrated higher activity than that of 5-FU against MCF-7. Compound **6,** which is a bis-indole alkaloid, is well known for its cytotoxic activity by inhibiting polymerisation of tubulin while compound **66**, which is one of the sphingolipids, was the most active against all four tested cell lines. The high activity of both compounds could be associated with their ability to cause cell cycle arrest and apoptosis by modulation of protein kinases and other signalling pathways. Meanwhile, compound β-Sitosterol 3-O-β-D-glucoside (**49)** was third in its activity against the HCT-116. The cytotoxicity of this compound was previously reported as moderate against HCT-cell and less so against MCF-7 cells compared to doxorubicin (Elsbaey et al., 2017).

The antiproliferative activity of all lignanamides compounds **26**–**30** and monoindole compounds (**11–19**) isolated from chloroform extract of *A. macrorrhiza* tuber against three human cancer cell lines, namely nasopharyngeal cancer (CNE-1), gastric cancer (MGC-803), and breast cancer (MCF-7). Based on the results, four compounds, benzodihydrofuran-type lignanamides (**26** and **29**) and monoindole compounds (**12** and **13**) displayed moderate antiproliferative activity against all three cancer cell lines, whereas the remaining compounds exhibited weak antiproliferative activity with IC_50_ values of larger than 100 µM. Huang et al. (2017b) also conducted a cytotoxic study of six piperidine alkaloids (**20–25**) isolated from the rhizomes of *A. macrorrhiza* (L.) Schott against five human cancer cell lines (CNE-1, Detroit 562, Fadu, MGC-803, and MCF-7) using the MTT method. Only one compound (**22**) exhibited cytotoxic effects against the four tested cell lines with IC_50_ values of less than 10 µM. The results indicated that a (2*S*, 3*R*, 6*R*)-2*-*methyl-3-hydroxy-piperidine skeleton could be conductive to cytotoxicity while the presence of carbonyl at the long-chain saturated carbonyl went against potent cytotoxicity. Furthermore, the presence of 4-OH at the piperidine skeleton showed no influence on the resultant potency. Hence, piperidine alkaloids could be responsible for the antiproliferative activity of *A. macrorrhiza* (Huang et al., 2017a).

The *in vivo* anti-malignant melanoma activity of 50% of ethanolic extract of *A. cucullata* (EAC) tuber showed the activity *via* modulation of the phosphatase and tensin homologue/phosphoinositide 3-kinase/AKT (PTEN/PI3K/AKT) pathway. Basically, PTEN is a tumour-suppressor gene and the loss of PTEN expression would activate the PI3K/AKT signalling pathway, which is responsible for the development and progression of tumours. Altered PTEN genes are found in various human cancers, including melanoma. PTEN is considered a major negative regulator of the PI3K/AKT signalling pathway, which modulates many cellular processes including cell proliferation, migration, invasion, and apoptosis, as well as tumour formation and progression (Fang et al., 2018). *In vitro* study found that EAC (concentrations between 20 and 80 μg/ml) suppressed the proliferation, migration, and invasion of murine melanoma cells (B-16) and human melanoma cells (A375 and A2058) in a time- and dose-dependant manner. In addition, the *in-Vivo* study demonstrated that EAC suppressed the growth of tumour in B16-bearing mouse in which a high dose (80 g/kg/day) exhibited a significant reduction in tumour volume and average weight as compared to low dose (0.5 g/kg/day) and medium dose (2 g/kg/day). The anti-tumour melanoma activity of the EAC, especially with a high dose (8 g/kg/day) was associated with the ability of EAC to increase the expression of PTEN and reduce the phosphorylation of PI3K and AKT *in-Vivo* and *in-Vitro* following the EAC treatment. Therefore, PTEN/PI3K/AKT may serve as a potential signalling pathway to target melanoma drug development. Summary of the anticancer activities of *Alocasia* species is presented in Table 4.

**TABLE 4:** Anticancer activity of *Alocasia* species.

Species	Compounds	Extract	Bioactivity	Methodology	Dosage/concentration	References
*A. cucullata (Lour.) G.Don*		Water extract of the root	Active in tumor developed from breast cancer cells (4T1 by reducing the tumor volumes, increasing the survival time of the mice, increasing the spleen size which subsequently increased cytokines production IFN-γ, IL-2 and TNF-α	*In vivo*	Tumour volumes (mg) at 5 different treatment conditions	Cai et al., (2013)
					Control groups: about 160 mg	
					Positive control: 100 mg	
					4 g/kg/day bw.: about 140 mg	
					8 g/kg/day bw.: about 130 mg	
					16 g/kg/day bw. about 96 mg	
					Survival time of the melanoma-bearing mice	
					8 g/kg/day bw water extract: 43 days	
					Negative control mice: 27 days	
					Weight index of spleen and thymus at 5 different treatment conditions	
					Control groups: about 65 and 20 respectively	
					Positive control: about 65 and 20 respectively	
					4 g/kg/day b.w.: about 70 and 23 respectively	
					8 g/kg/day b.w.: about 79 and 25 respectively	
					16 g/kg/day b.w. about 82 mg and 20 respectively	
					Levels of IL-2, IFN-γ and TNF-α at 5 different treatment conditions	
					Control groups: about 165, 40 and 120 ng/ml respectively	
					Positive control: about 210, 60 and 140 ng/ml respectively	
					AC at 4 g/kg/day b.w.: about 160, 35 and 120 ng/ml respectively	
					AC at 8 g/kg/day b.w.: about 165, 60 and 120 ng/ml respectively	
					AC at 16 g/kg/day b.w. about 210, 60 and 170 ng/ml respectively	
		Water extract of the root	The *Alocasia cucullata* extract induced differentiation of cultured human monocytic cell lines, THP-1 in a dose-dependent manner and increased their cytokines production (IL-1β and TNF-α)	*In vitro*	% of total adherent cell at 5 different treatment condition	Cai et al., (2013)
					Control groups: 1%	
					AC at 250 mg/L: about 3%	
					AC at 500 mg/L: about 10%	
					AC at 1000 mg/L: about 40%	
					AC at 2000 mg/L: about 45%	
					PMA (10 ng/ml): about 95%	
					IL-1β and TNF-α expression of THP-1 cells treated with *A. cucullata* extract at concentration of 2 mg/ml	
					TNF-α: about 51 pg/ml, 18 pg/ml and 22 pg/ml after 24, 48 and 72 h after AC treatment respectively	
					IL-1β: about 18 pg/ml and 38 pg/ml after 24 and 48 h after AC treatment respectively	
	(**52**), (**53**), (**54**), (**55**), (**56**) and (**60**)	Ethanol extract of tuber	All the compounds showed effective cytotoxic activities against gastric cancer cell line (MGC-803 cell line) and colon cancer cell line (HT-29 cell line)	*In vitro*	IC_50_ of the compounds against MGC-803 and HT-29 cell lines	Peng et al. (2016)
					Compound (**54**): 0.98 ± 0.3 μg/ml and 1.56 ± 0.8 μg/ml, respectively	
					Compound (**52**): 29.3 ± 0.2 μg/ml and 41.6 ± 0.7 μg/ml, respectively	
					Compound (**53**): 32.9 ± 0.3 μg/ml and 39.5 ± 1.1 μg/ml, respectively	
					Compound (**55**): 33.6 ± 0.3 μg/ml and 38.4 ± 1.3 μg/ml, respectively	
					Compound (**60**): 45.6 ± 0.2 μg/ml and 28.4 ± 1.1 μg/ml, respectively	
					Compound (**56**): 25.6 ± 0.3 μg/ml and 32.4 ± 0.2 μg/ml, respectively	
		Ethanol extract of tuber, further partitioned with butanol	Exhibited active anti-proliferative activity against 4 cancer cell lines; gastric cancer (MGC-803), breast cancer (MDA-MB-435), myelogen leukemia cancer (K-562), Liver carcinoma (Bel7402) in dose-dependent manner except cervical cancer (Hela)	*In vitro*	IC_50_ value of EAC-B at 48-h on different cell lines	Wei et al. (2015)
					MGC-803: 24.1 ± 3.7 μg/ml	
					MDA-MB-435: 26.0 ± 2.7 μg/ml	
					K-562: 27.9 ± 7.8 μg/ml	
					Bel7402: 39.9 ± 6.1 μg/ml	
					Hela >50 μg/ml	
		Ethanol extract of tuber, further partitioned with butanol	Inhibited tumor growth and increased necrosis in mice-treated MGC-803 cells in a dose-dependent manner	*In vivo*	1 g/kg and 5 g/kg	Wei et al. (2015)
	Alocasgenol (**53**)	Ethanol extract of tuber, further partitioned with butanol	Alocasgenol (35a), and alocasgenoside B (37) strongly inhibit the growth of gastric cancer (MGC-803) and colon cancer cells (HT-29)	*In vitro*	IC_50_ value of compounds ( **53** ) and (37) at 48-h on different cell lines	(Wei et al., 2015)
	Alocasgenoside B (**54**)				Alocasgenol (**53**): 32.9 ± 0.3 μg/ml (MGC-803), 39.5 ± 1.1 μg/ml (HT-29)	
					Alocasgenoside B (**54**): 0.98 ± 0.3 μg/ml (MGC-803), 1.56 ± 0.8 μg/ml (HT-29)	
	N-acetyl-D-lactosamine (LacNAc) (**50**)	Purified from phosphate buffered saline supernatant using asialofetuin-linked amino activated silica	Significant anti-proliferation activity against cervical cancer cell line (SiHa) and prostate cancer cell line (PC-3), but poor anti-proliferative activity against central nervous system (SNB-78) and breast cancer cell line (A-549)	*In vitro*	% growth inhibition of *A. cucullata* extract at concentration of 100 μg/ml: (SiHa): 50%	Kaur et al. (2005)
					(PC-3): 16%	
					(SNB-78): 23%	
					(A-549): 46%	
		50% Ethanol extract of tuber	Suppressed proliferation, migration, and invasion of melanoma skin cancer cells (B16-F10, A375 and A2058) in a dose-dependent manner (0, 5, 10, 20, 40, and 80 µg/ml) by modulating PTEN/PI3K signaling/AKT	*In vitro*	Cell viabilities at *A. cucullata* concentrations of 0, 5, 10, 20, 40, and 80 μg/ml (at 24 h)	Fang et al. (2018)
					B16-F10: 100%, 88.84 ± 3.32%, 86.88 ± 2.6%, 80.01 ± 4.01%, 67.31 ± 2.99% and 56.53 ± 2.14%	
					A375: 100%, 97.44 ± 5.12%, 92.84 ± 5.08%, 82.94 ± 2.55%, 79.23 ± 3.39% and 76.99 ± 5.27%	
					A2058: 100%, 97.11 ± 2.52%, 95.80 ± 2.13%, 92.84 ± 2.95%, 89.72 ± 2.68% and 86.96 ± 4.39%	
					Cell viabilities at *A. cucullata* concentrations of 0, 5, 10, 20, 40, and 80 μg/ml (at 48 h)	
					B16-F10: 100%, 87.96 ± 3.74%, 81.49 ± 4.35%, 63.7 ± 4.53%, 47.77 ± 3.34% and 40.98 ± 4.4%	
					A375: 100%, 97.35 ± 1.79%, 80.59 ± 2.45%, 76.50 ± 2.45%, 75.23 ± 2.88% and 71.45 ± 3.45%	
					A2058: 100%, 94.72 ± 3.64%, 93.57 ± 4.63%, 86.56 ± 4.49%, 80.86 ± 3.17% and 78.14 ± 5.37%	
					IC_50_ values of melanoma cells at 24- and 48-h	
					B16-F10: 63.35 and 35.06 μg/ml respectively	
		50% Ethanol extract of the tuber	Reduce the average volume and weight of B16-F10 melanoma-bearing mice. The extract also effectively increased PTEN level and decreased AKT levels in xenografted B16-F10 tumors in mice	*In vivo*	Average tumor volume	Fang et al. (2018)
					Control group: 2271.51 ± 386.44 mm^3^	
					0.5 mg/kg/day of EAC: 1857.44 ± 365.29 mm^3^	
					2 mg/kg/day of EAC: 1143.17 ± 296.15	
					8 mg/kg/day of EAC: 807.55 ± 241.67 mm^3^	
					Average tumor weight	
					Control group: 2.56 ± 0.35 g	
					0.5 mg/kg day of EAC: 2.13 ± 0.34 g	
					2 mg/kg day of EAC: 1.41 ± 0.36	
					8 mg/kg day of EAC: 1.01 ± 0.34 g	
					PTEN and phosphorylated AKT levels in	
					Control groups: 11.05 ± 14.51 and 114331.87 ± 4957.85 respectively	
					0.5 g/kg/day EAC: 873.37 ± 1067.81 and 94087.68 ± 9672.48 respectively	
					2 g/kg/day EAC: 1321.13 ± 1231.07 and 32141.84 ± 4028.88 respectively	
					8 g/kg/day EAC: 2690.22 ± 1040.04 and 17493.15 ± 2145.72 respectively	
*Alocasia macrorrhiza*	Hyrtiosulawesin (7), Alocasin A (1), Alocasin B(2), Alocasin D(4), Alocasin E (5)	Ethanol extract of therhizome	Hyrtiosulawesin (**7**), Alocasin A (**1**), Alocasin D (**4**), and Alocasin E (**5**) exhibited mild antiproliferative activity against Hep-2 and Hep-G2. Hyrtiosulawesin (**7**), and Alocasin B (**2**) showed antiproliferative activity against nasopharyngeal cancer (CNE)	*In vitro*	IC_50_ of compounds (7), (1), (4) and (5) against Hep-2 are 35, 151, 132, and 122 μM, respectively	Zhu et al. (2012)
					IC_50_ of compounds (7), (1), (4) and (5) against Hep-G2 are 189, 85, 136, and 193 μM respectively	
					IC_50_ of compounds (7) and (2), against CNE are 55 and 137 μM, respectively	
	(2*S*,3*R*,6*R*)-2-methyl-6-(9- phenylnonyl) piperidin-3-ol (**23**)	Chloroform extract of the rhizome	Compound **23** exhibited cytotoxicity activity against Detroit 562, Fadu, MGC-803, and MCF-7 human cancer cell lines under MTT assay. Cisplatin was used as positive control	*In vitro*	IC_50_ of compound (19) against Detroit 562, Fadu, MGC-803, and MCF-1 cell lines were all less than 10 µM	Huang et al. (2017a)
					IC_50_ of cisplatin against CNE-1, Detroit 562, Fadu, MGC-803, and MCF-1 cell lines were 6.8 ± 2.5, 7.4 ± 0.4, 6.5 ± 0.6, 5.8 ± 0.8 and 15.9 ± 1.6	
	All compounds lignanamides (**19**–**23**) and monoindole compound (**11**–**19**)	Chloroform extract	(**12** and **15**) showed moderate antiproliferative activity against the three cancer cells (nasopharyngeal cancer (CNE-1), gastric cancer (MGC-803), and breast cancer (MCF-7)), whereas the other compounds exhibited weak antiproliferative activity with IC_50_ values larger than 100 µM	*In vitro*	IC_50_ (µM.) of compounds 12, 15, 47, 48 and cisplatin against	Huang et al. (2017a)
					CNE-1: 85.91 ± 10.10, 72.98 ± 20.82, 63.10 ± 5.43, 6.83 ± 2.49 respectively	
					MGC-803: 80.20 ± 8.67, 112.25 ± 2.68, 32.84 ± 4.23, 73.50 ± 1.56, 5.79 ± 0.82 respectively	
					MCF-7: 96.08 ± 13.08, 85.24 ± 4.75, 31.16 ± 7.29, 31.16 ± 7.29, 64.55 ± 5.01, 15.94 ± 1.57, respectively	
		Water extract of the tuber	Exhibits proliferation inhibition and apoptotic effects on human hepatocellular carcinoma cells (SMMC-7721) under MTT assay. Human normal liver cell (L02) was used as negative control	*In vitro*	Cell viability (%) of L02 and SMMC-7721 after 5 days at different concentrations of water-soluble AME	Fang et al. (2012)
					100 μg/ml: 98% and 89% respectively	
					200 μg/ml: 92% and 85% respectively	
					300 μg/ml: 90% and 75% respectively	
					400 μg/ml: 80% and 45% respectively	
					500 μg/ml: 72% and 21% respectively	
					Apoptotic effects on L02 and SMMC-7721 after 48 h at 400 μg/ml of water-soluble AME	
					L02 control: 2.2 ± 1.8	
					L02 with 400 μg/ml AME: 1.3 ± 1.6	
					SMMC-7721 control: 2.0 ± 0.5	
					SMMC-7721 with 400 μg/ml AME: 6.4 ± 0.9	
		Water extract of the tuber	Inhibits the growth of murine hepatoma (H22) cells in murine hepatoma-bearing mice	*In vivo*	Mean tumor weight of mice after 10 days	Fang et al. (2012)
					Control mice: 1.01 ± 0.43 g	
					Mice with 0.8 g/kg/day of water-soluble MAE: 0.61 ± 0.46 g	
					Mice with 0.4 g/kg/day of water-soluble MAE: 0.94 ± 0.63 g	
					Mice with 0.2 g/kg/day of water-soluble MAE: 0.95 ± 0.43 g	
					Tumor inhibitory rates of different concentrations of water soluble AME	
					0.8 g/kg/day of water-soluble MAE: 40.20%	
					0.4 g/kg/day of water-soluble MAE: 7.84%	
					0.2 g/kg/day of water-soluble MAE: 6.86%	
	Hyrtiosin B (6), 1-O-β-D-glucopyranosyl-(2*S*, 3*R*, 4*E*, 8*Z*)-2-[(2(*R*)-hydroctadecanoyl) amido]-4,8-octadecadiene-1,3-diol (**58**), 3-epi-ursolic acid (**47**), 3-epi-betulinic acid (**48**), β-sitosterol (**46**), β-sitosterol 3-O-β-D-glucoside (**49**)	Methanol extract of the rhizome	The total extract was cytotoxic against the human larynx cancer cell line (Hep-2) and colon cancer cell (HCT-166) and less so against HepG2 and MCF-7 cell lines. Compounds (6), (**58**) and (**49**) isolated from the extracts have remarkable cytotoxic activity	*In vitro*	IC_50_ values of the total extract against Hep-2, HCT-116, HepG2 and MCF-7 cell lines are about 7, 8, 16 and 18 μg/ml respectively as compared to 5-FU which (IC_50_ of 5, 6, 15, 17 μg/ml, respectively)	Elsbaey et al. (2017)
					IC_50_ values of isolated compounds against Hep-2, HCT-116, HepG2 and MCF-7 cell lines	
					Compound (**6**): about 28, 15, 19 and 34 μg/ml respectively	
					Compound (**65**): about 23, 10, 12 and 23 μg/ml respectively	
					Compound (**41**): about 62, 25, 25 and 58 μg/ml respectively	
					Compound (**46**): about 57, 23, 26 and 54 μg/ml respectively	
					Compound (**47**): about 41, 26, 19 and 45 μg/ml respectively	
					Compound (**49**): about 54, 31, 16 and 53 μg/ml respectively	
					5-FU: about 62, 40, 49 and 39 μg/ml respectively	
		50% ethanol extract of the rhizome	Significant inhibitory effect on gastric cancer cells M-GC803	*In vitro*	IC_50_ = 121 μg/ml	(Lei X, Feng Y, Liang S, Wang Y, Zheng X, 2012)

a w = body weight; p.o = oral route.

### Antidiabetic and Antihyperglycaemic Activity

Diabetes mellitus is a metabolic disorder characterised by prolonged high blood sugar level in the body (hyperglycaemia) with impaired metabolism of carbohydrates, lipids, and proteins due to defects in insulin secretion or insulin activity or both (American Diabetic Association, 2008). An increase in blood sugar level can increase oxidative stress and production of free radicals, consequently contributing to the progression of diabetic complications. Moreover, diabetic patients possessed lesser antioxidants such as vitamin C and vitamin E, or lower activities of antioxidant enzymes such as catalase, superoxide dismutase (SOD), and glutathione peroxidase compared to normal people. It was believed that flavonoids-rich plants are useful to control diabetes as the compounds possess potent ROS scavenging activity (Patil et al., 2012; Karim et al., 2014).

A phytochemical screening of *Alocasia* plant extracts mostly showed the presence of flavonoids, alkaloids, and steroids (Patil et al., 2012; Karim et al., 2014; Jawaid et al., 2015) which may contribute to their traditional uses in managing lipidemia and diabetes (Patil et al., 2012). Further study using the purified active principles from parts of the plant extracts may reveal the role of the respective preparations as hypoglycaemic agents in diabetes management. Meanwhile, Streptozotocin (STZ) and alloxan are commonly used in most rat experimental models to induce diabetes and hyperglycaemia in rats as they can selectively kill pancreatic β-cells and impair insulin secretion (Karim et al., 2014; Jawaid et al., 2015). In this review, *A. macrorrhiza* (Linn.) and *A. indica* (Roxb.) Schott was reported to possess antidiabetic and antihyperglycaemic activities.

The ethanol extracts of *A. indica* L. leaves and stem were effective on STZ-induced rats at doses ranging between 200 and 400 mg/kg in a dose-dependent manner (Karim et al., 2014). Patil et al. (2012) also used alcohol extract of *A. indica* (Roxb.) leaves in STZ-induced diabetic rats at concentrations of 200 and 400 mg/kg BW with glibenclamide as the positive control. The results showed decreased blood glucose levels and serum lipid profiles such as cholesterol and triglyceride in the tested animals. Both studies suggested that the *Alocasia* leaves and stem extracts were able to stimulate pancreatic β-cells to release insulin, which was similar to that of the sulfonylurea drug glibenclamide (Karim et al., 2014; Jawaid et al., 2015). Another possible mechanism of action of the plant extracts includes the increase in the re-uptake of glucose by liver cells, thus inhibiting gluconeogenesis and activates insulin receptors (Karim et al., 2014).

The ethanol extract of *A. indica* rhizomes at doses between 100 and 200 mg/kg BW exhibited a significant decrease in blood glucose level, serum total cholesterol, triglycerides, low-density lipoprotein (LDL), and very-low-density lipoprotein (VLDL). The results also indicated an increase in high-density lipoprotein (HDL) in two rat models; High Fat Diet/Streptozotocin (HFD/STZ)- and Streptozotocin/Nicotinamide (NTZ/Nicotinamide)-induced lipidemia and type 2 diabetes, rat models. In contrast, Rahman et al. (2012) found that the methanolic extract of *A. macrorrhiza* rhizome used in alloxan-induced hyperglycaemic mice at a concentration of 250 mg/kg and 500 mg/kg exhibited a dose-dependant decrease in blood glucose level with significant activity at 500 mg/kg dose compared to metformin (150 mg/kg). In addition, the blood glucose level at a dose of 250 mg/kg and 500 mg/kg recorded 41.70% and 55.49% reduction at 8 h of treatment compared to the diabetic control (Packer et al., 2012).

### Antioxidant Activity

Herbal extracts with antioxidant properties are effective against oxidative stress caused by ROS and health disorders such as cancer, diabetes, ageing, and hepatic damage as they can block the formation of free radicals (Mulla et al., 2009a; Rahman et al., 2012; Abdulhafiz et al., 2020). The hydroxyl radical (OH^·^) is the most reactive ROS that can cause severe cell or tissue damage. Nitric oxide (NO), which is produced from sodium nitroprusside in various physiological processes, reacts with oxygen to form nitrite (NO_2_^−^). The overproduction of NO is always related to disease conditions such as carcinomas and inflammation. Superoxide radical (O_2_^−^) is also detrimental to biological systems due to its ability to break down and form more powerful oxidative species such as singlet oxygen and OH^**·**^ (Pal et al., 2014a; Phaniendra et al., 2015). All these harmful free radicals cause enzyme inactivation and cellular components impairment *via* covalent binding and lipid peroxidation, consequently leading to serious tissue injury (Mulla et al., 2009b; Pal et al., 2014b).

The presence of natural antioxidant enzymes as free radical scavengers in plants such as catalase, SOD, and glutathione peroxidase are crucial to resist oxidative stress (Mulla et al., 2009a; Pal et al., 2014a) by suppressing and inhibiting the formation of free radicals as well as inhibiting lipid peroxidation (Mulla et al., 2009b). In addition, plant extracts that are rich in antioxidant bioactive molecules such as phenolic, flavonoids, and alkaloids are capable of removing free radical intermediates from the body and block the progression of chain reactions, thus, preventing cell damage and disease development. For instance, phenolic compounds can neutralise lipid free radicals and prevent the decomposition of highly reactive species, while flavonoids can scavenge different ROS including hydrogen peroxide (H_2_O_2_), OH^−^, peroxyl (HO_2_), and O_2_^−^ (Islam et al., 2013).

The compound 2,2-diphenyl-1-picrylhydrazyl (DPPH) contains stable free radicals, which is normally used to evaluate the antioxidant potency of plant extracts (Abdulhafiz et al., 2020), especially plants that are rich in phenolic compounds and flavonoids (Mulla et al., 2009a; Rahman et al., 2012; Islam et al., 2013). The bioactive compounds can convert the highly reactive free radical in DPPH into stable non-reactive DPPH form by donating hydrogen to a free radical, thus removing odd electrons that are responsible for the reactivity of free radicals (Islam et al., 2013; Pal et al., 2014a). The scavenging activity of plant extracts is expressed as IC_50_ (µg/ml), which is the concentration of the sample required to scavenge 50% of DPPH free radical (Mandal et al., 2010).

Previously, the antioxidant activity of hydroalcoholic extract of *A. indica* (Linn.) leaves was investigated by (Mulla Wahid et al., 2010a) using several antioxidants models including DPPH, NO, OH^−^, and superoxide free radical scavenging assays. The results showed that all different concentrations of the extract (50, 100, 200, 400, 800, and 1000 μg/ml, respectively) possessed potent antioxidant activity in a dose-dependant manner and were comparable to that of the standard ascorbic acid at 200 μg/ml. In addition, the extract showed better activity in quenching NO radical and DPPH with IC_50_ values of 9.69 and 9.15 μg/ml, respectively and moderate activity in the remaining antioxidant assays. At 1000 μg/ml, the extract showed maximum scavenging of superoxide radical (87.17%), followed by stable DPPH (83.48%), NO radical (74.09%), and hydroxyl radical (60.96%) (Mulla et al., 2009b). Mulla et al. further studied the antioxidant activity of ethanol extract of *A. indica* leave in the same antioxidant assays with the same range of concentrations. The results showed that the extract exhibited potent antioxidant activity in a dose-dependant manner with the maximum inhibitory concentration (IC_50_) in all models were 7.30, 10.97, 9.8, and 7.86 μg/ml respectively (Mulla Wahid et al., 2010b).

The antioxidant property of *A. macrorrhiza* (rhizome, root, and leave) and *A. fornicata* (stolon and leave) in different solvent extracts (hexane, benzene, toluene, chloroform, diethyl ether, ethyl acetate, and aqueous fraction) were conducted in comparison to standard antioxidants (quercetin and ascorbic acid) using the DPPH assay. The results showed that the IC_50_ values of some solvent extracts were less than that of the standards. The quercetin and ascorbic acid recorded an average IC_50_ value of 78.17 ± 4.05 and 53.60 ± 1.79 μg/ml, respectively. The maximum antioxidant activity of diethyl ether of rhizome and root of *A. macrorrhiza* were 48.01 ± 6.68 and 34.51 ± 2.71 μg/ml, respectively. Meanwhile, for *A. fornicata*, only diethyl ether of stolon and leave showed maximum antioxidant activity of less than the standards with IC_50_ values of 31.11 ± 7.02 and 41.23 ± 9.44 μg/ml, respectively. This study suggested that the extraction of bioactive antioxidants from rhizomes, roots, and stolon of aroids is not suitable using highly hydrophobic solvents such as hexane or polar solvents such as water. However, the water extract of the plant leaves may be capable of producing optimum antioxidant activity that is comparable to the standards (Mandal et al., 2010).

The antioxidant activity of ethanol extract of *A. indica* tuber (Islam et al., 2013) and *A. macrorrhiza* rhizome (Rahman et al., 2012) was evaluated through the DPPH radical scavenging assays. Based on the results, both the extract and ascorbic acid showed a gradual increase in scavenging activity at a lower concentration. In contrast, a plateau was achieved at a higher concentration, particularly at a concentration of approximately 128 μg/ml (Islam et al., 2013) and 100 μg/ml (Rahman et al., 2012). Moreover, the IC_50_ values for both plant extracts were 42.66 μg/ml and 693.0 μg/ml in the former and latter studies, respectively, indicating that the ethanol extract of *A. indica* tuber was more potent than that of the ethanol extract of *A. macrorrhiza* rhizome.

The DPPH free radical scavenging activity of the methanol extract of *A. macrorrhiza* roots and its different soluble fractions (carbon tetrachloride, petroleum ether, chloroform, and aqueous) reported that the antioxidant activity of all the extracts increased with increasing concentration. Moreover, the methanol extract showed the highest free radical scavenging activity with IC_50_ values of 47.11 μg/ml, followed by moderate antioxidant activity from petroleum ether fraction (IC_50_ = 65.04 ug/mL), carbon tetrachloride fraction (IC_50_ = 107.34 μg/ml), aqueous fraction (IC_50_ = 170.13 mg/ml), and chloroform fraction (IC_50_ = 201.39 μg/ml) (Banik et al., 2014).

The DPPH scavenging activity of the ethanol extract of the *A. indica* tuber was 3.5 times higher compared to the aqueous extract. The ethanol extract also exhibited higher OH^−^, NO, and superoxide radicals scavenging activity in comparison to the aqueous extract. In addition, the study found that ethanol extract contained higher antioxidant enzymes (SOD and the protein catalase), phenolic, and flavonoids contents compared to those in the aqueous extract, which explained its higher potent antioxidant activity (Pal et al., 2014b.

Besides the ability of *Alocasia* plant extracts to donate hydrogen ions from antioxidant bioactive molecules to the DPPH free radicals to form inactive stable DPPH molecules, it was believed that the possible mechanism of action by the plant extracts involves the competition with oxygen molecules to react with NO radicals in order to inhibit the generation of nitrite. In fact, the formation of O_2_^·−^ (the first reduction product of oxygen) and OH^−^ radical can also be inhibited (Mulla et al., 2009a; Mulla Wahid et al., 2010a; Pal et al., 2014b). Therefore, it was suggested that the antioxidant properties *Alocasia* plant is a combined contribution from phenolic compounds, flavonoids, alkaloids, and other constituents found in the extracts (Mulla et al., 2009b; Mulla Wahid et al., 2010b; Mandal et al., 2010; Pal et al., 2014a; Banik et al., 2014).

### Antidiarrhoea Activities

Diarrhoea is a condition where the excessive passage of watery stools occurred due to an increase in bowel movements, impaired intestinal absorption, and excessive intestinal secretion of water and electrolytes (Mulla Wahid et al., 2011; Islam et al., 2013). It is a common illness and a major public health concern in developing countries, especially among people and communities with poor standards of hygiene (Mulla Wahid et al., 2011). *Alocasia* plant extracts exhibit antimotility, antimicrobial, and antisecretory effects, which could be contributed by the presence of flavonoids, alkaloids, sterols, terpenes, and other constituents (Mulla Wahid et al., 2011).

The castor oil- and magnesium sulphate-induced diarrhoea models are commonly used to evaluate the antidiarrhoea activity of plant extracts (Mulla Wahid et al., 2011; Islam et al., 2013). The secretory diarrhoea, which is associated with hypersecretory response, is a type of diarrhoea triggered by ricinoleic acid, an active metabolite of castor oil (Mulla Wahid et al., 2011). The ricinoleic acid induces diarrhoea by enhancing the peristaltic movement of the small intestine and secretion of intestinal content, which is due to the release of prostaglandins while magnesium sulphate triggers diarrhoea by inhibiting the reabsorption of fluids and electrolytes, subsequently leading to a build-up in intestinal content. In addition, magnesium sulphate increases intestinal motility by releasing cholecystokinin from the duodenal mucosa (Mulla Wahid et al., 2011; Islam et al., 2013).

The *in-Vitro* and *in-Vivo* antidiarrhoea activity of aqueous and ethanol extracts of *A. indica* leaves showed that the aqueous and ethanol extracts of the plant exhibited significant growth inhibition on diarrhoea-caused microbes (*E. coli*, *S. typhimurium*, *S. flexneri*, and *S. aureus*) *in-Vitro* in a dose-dependant manner. The ethanol extract also exhibited a larger zone of inhibition diameter than that of aqueous extract of the plants. The *in-Vivo* studies suggested that both extracts of *A. indica* exhibited antidiarrhoea activity by increasing the absorption of water and electrolyte from the gastrointestinal tract. The reduction in the peristaltic index in ricinoleic acid-induced intestinal transit study also suggested that extracts of *A. indica* activate the sympathetic innervations of the intestine which inhibited peristaltic activity and tone reduction. Moreover, the significant reduction in the weight and volume of intestinal contents was associated with the blocking of intraluminal fluid accumulation induced by ricinoleic acid in a dose-dependant manner. All extracts were found to alleviate the diarrhoeic condition in all models similar to the loperamide with the most significant effect was exhibited by the ethanol extract at 400 mg/kg (Mulla Wahid et al., 2011).

Furthermore, Islam et al. (2013) studied the *in-Vivo* antidiarrhoea activity of ethanol extracts of *A. indica* tuber in both castor oil- and magnesium sulphate-induced diarrhoea mice models. Based on the study, the plant extracts were able to prevent diarrhoea by decreasing gastrointestinal motility by 38.90% at a concentration of 250 and 500 mg/kg BW in addition to the inhibition of bowel movements of approximately 56.34%. It was suggested that the antimotility activity of the plant extracts was due to its ability to increase reabsorption of aqueous substances and electrolytes, thus, increasing the intestinal content.

### Antimicrobial and Antifungal Activity

The antimicrobial activity of plant species is normally determined using the disk diffusion method which measures the zone of inhibition of the plant extracts against gram-positive and gram-negative bacteria (Haque et al., 2014). The factors that influence the size of the inhibition zone include the capability of the substances in the plant extract to diffuse through the medium as well as the metabolic activity and growth of microorganisms in the medium (Haque et al., 2014). In addition, the lipid content of the membranes of the different bacterial groups and the permeability of various constituents of the plant extracts would influence the size of the inhibition zone (Roy et al., 2013). The ability of plant extracts to demonstrate antimicrobial activity against both gram-positive and gram-negative could indicate the presence of a broad-spectrum of antibiotic compounds (Haque et al., 2014).

An antimicrobial study on different extracts of *A. indica* leaves, including petroleum ether, chloroform, acetone, ethanol, and water against several microorganism exhibited significant antimicrobial activities against gram-positive and gram-negative bacterial as well as fungal strains. In addition, the Minimum Inhibitory Concentration (MIC) values of all extracts were reported between 5 and 20 mg/ml. The lowest and highest MIC values observed from the ethanol and chloroform extracts of the plant ranged between 10.23 and 13.18 mg/ml and 14.30–15.42 mg/ml, respectively. The inhibition zone of all extracts increased with increasing concentrations, ranging between 9 and 23 mm (Mulla Wahid et al., 2010a).

The study also showed that ethanol extract (10 mg/ml) recorded the most significant inhibitory activity against *B. subtilis* with an inhibition zone of 22 mm, followed by *E. coli* (19 mm), *S. cerevisiae* (18 mm), *K. pneumonia* (17 mm), *S. aureus* (16 mm), *C. albicans* (16 mm), and *A. niger* (15 mm). The extract inhibited the growth of bacteria compared to that of antibacterial standard (Gentamicin 0.5 mg/ml) but exhibited less inhibitory activity against fungi strains compared to the standard antifungal (fluconazole 0.5 mg/ml). This study suggested that bioactive compounds found in *A. indica* leave extracts such as polyphenolics compounds such as tannin played a major role in antimicrobial activity. Tannin in the form of crude extract is mostly tested compared to individual compound against microorganism. Tannin acts as antimicrobial by interacting with bacterial enzymes and precipitating them (Puljula et al., 2020).

In another study, Islam et al. (2013) used the ethanol extract of *A. indica* Schott tuber to evaluate its antimicrobial activity against 2 g-positive bacteria (*S. aureus and Staphylococcus epidermidis*)*,* 6 g-negative bacteria (*S. typhi, E. coli, S. flexneri*, *Shigella sonnei*, *Shigella dysenteriae*, *and K. pneumonia*), and three fungal species (*A. niger*, *C. albicans*, and *S. cerevisiae*)*.* The results revealed that the plant extracts demonstrated moderate antimicrobial activity with an inhibition zone ranging between 5.8–9.8 mm and 12.1–18 mm for gram-positive and gram-negative bacteria, respectively when the concentration was set at 250 and 500 µg/disc.

The evaluation of antimicrobial activity of *A. macrorrhizos* extracts (petroleum ether, carbon tetrachloride, chloroform, and aqueous fraction) at 400 µg concentration/disk against *B. subtilis*, *S. aureus*, *Pseudomonas aeruginosa*, *S. typhi*, *E. coli*, *C. albican*s, and *A. niger* showed that the methanol crude extract was effective against all tested microorganisms while the chloroform soluble fractions were selectively effective against all tested gram-negative bacteria only. In addition, the carbon tetrachloride soluble fractions were effective against all gram-positive and gram-negative bacteria, but not against fungi. In contrast, petroleum ether fraction was effective against all bacteria except *S. areus* and *P. aeruginosa* while the aqueous soluble fraction effective except against *S. typhi.* This study suggested that certain chemical constituents found in different parts of plant extracts may be responsible for their antimicrobial activity against certain types of microorganisms. Thus, further study is needed to detect the chemical compounds that exert the highest antimicrobial and antifungal activities (Banik et al., 2014).

Furthermore, Haque et al. (2014) examined the antimicrobial activity of *A. fornicata* leaf, stolon, and root extracts using ethanol and other soluble partitions (petroleum ether, chloroform, and ethyl acetate) against *Bacillus megaterium*, *B. subtilis*, *Bacillus cereus*, *S. aureus*, *Sarcina lutea*, *Salmonella paratyphi*, *Vibrio parahaemolyticus*, *Vibrio mimicus*, *E. coli*, *S. dysenteriae*, *P. aeruginosa*, *and Shigella boydii.* The results showed that all extracts (500 µg/disk) exhibited good antibacterial effect except for the petroleum ether extracts which did not record any antimicrobial activity. The ethanol extract of plant root was the most active against all the bacteria with a zone of inhibition ranging between 10 and 18 mm. Meanwhile, the chloroform and ethyl acetate leaves extracts were more active against most of the tested bacteria compared to the respective stolon extracts even though the chloroform extract of the stolon showed the highest zone of inhibition (20 mm) against *S. lutea.* Besides, both ethyl acetate and chloroform extracts of the plant leaves showed better MIC against *B. subtilis* at 64 μg/ml while ethanol extract of roots recorded a MIC of 64 μg/ml against *P. aeruginosa*.

The antimicrobial properties of methanol extract of *A. decipiens* Schott rhizome were investigated *in-Vitro* by Roy et al. (2013) against 2 g-positive bacteria (*S. aureus* and *B. subtilis*) and 2 g-negative bacteria (*E. coli* and *Klebsiella sp.*). The results showed a significant zone of inhibition against *S. aureus*, *B. subtilis, E. coli,* and *Klebsiella sp.* at 16 mm, 12 mm, 11 mm, and 10 mm, respectively when the extract was at 100% concentration. The MIC value of the extract was varied between 2 and 16 μg/ml with *S. aureus* displayed higher sensitivity while *Klebsiella sp.* was the most resistant bacteria towards the plant extracts. All the organisms were inhibited with the concentration of plant extracts at 25% except for *S. aureus* which was inhibited at 10% concentration. The methanol plant extracts were believed to have broad-spectrum activity against gram-positive bacteria.

A less effective antimicrobial effect was demonstrated by *A. sanderiana* Bull. leaves through three different solvent extracts (methanol crude extract, dichloromethane fraction of methanol extract, and hexane fraction of methanol extract) (Ongpoy Jr et al., 2015). The results showed that the antimicrobial activities of the plant leave extracts against 8 g-positive bacteria, 8 g-negative bacteria, and three fungi using at least an 8 mm inhibition zone was mostly non-active. However, some areas were observed below the 8 mm criteria, with the dichloromethane fraction displaying an inhibition zone of 4 mm, 3 mm, 1 mm, and 1 mm for *Proteus mirabilis*, *P. aeruginosa*, *Pectrobacterium carotovorum,* and *C. albicans*, respectively while methanol fraction showed an inhibition zone of 1 mm against *P. aeruginosa.* The study suggested that the polyphenolic compounds found in the plant extracts may not be an effective antimicrobial agent against all the microorganisms tested. Furthermore, the small zone of inhibition (less than 8 mm) exhibited by some of the plant extracts may be due to the presence of protease inhibitors, trypsin inhibitors or lectins in which their roles in antimicrobial activity still requires further analysis (Ongpoy Jr, 2015; 2017).

The antimicrobial study on 80% ethanol extract of *A. denudata* stem against selected gram-positive oral bacteria which include *S. mutans*, *S. aureus*, and *E. faecalis* as well as the non-oral pathogen *Streptococcus pyogenes* indicated the presence of the antimicrobial steroid compound β-sitosterol trimethylsilyl ether, the results recorded no antimicrobial effects by the plant extracts at any concentration even up to 32 μg/ml. In addition, compounds such as phenols, flavonoids, and alkaloids, which had been proven to possess antimicrobial effects, were not detected in the extract. It was believed that either the antimicrobial compounds were degraded before the susceptibility testing was performed or that the selected bacteria were already resistant to the compounds (Mohd Yusoff et al., 2020).

So far, seven *Alocasia* species had been studied for their antimicrobial and antifungal activities. Based on previous studies, *A. indica* syn. and *A. macrorrhizos* are the most studied species that showed significant antimicrobial effects comparable to that of standards. Meanwhile, *A. fornicata* and *A. decipiens* Schott showed moderate-to-good antimicrobial activities. In contrast, *A. sanderiana* Bull., *A. denudata*, and *A. brisbanensis* extracts exhibited no antimicrobial activities. Thus, it was believed that different extracts of the *Alocasia* species may contain different bioactive molecules that are responsible for antimicrobial and antifungal activity.

### Antiparasitic (Antiprotozoal and Anthelminthic) Activity

Several worm samples such as *Ascaridia galli*, *Ascaris lumbricoides*, and *Pheretima posthuma* are normally used to evaluate the *in-Vitro* antihelminthic activities of plant extracts. The most common test worm used in experiments is the *P. posthuma* due to its similar anatomical and physiological characteristics to the intestinal roundworm parasite of humans (Mulla Wahid et al., 2010b; Banik et al., 2014). The anthelminthic activity of *A. indica* (Roxb.) Schott leaves were evaluated by (Mulla Wahid et al., 2010a) using hydroalcoholic extract, petroleum ether fraction, and ethyl acetate fraction of the plant against *P. posthuma*. It was revealed that all the extracts (concentrations of 10, 25, and 50 mg/ml) were vermifuge and vermicidal in a dose-dependant manner. In addition, the use of hydroalcoholic extract at a concentration of 50 mg/ml was the most effective which took only 8 min and 14 min to paralyse and kill *P. posthuma*, respectively. The ethyl acetate fraction of *A. indica* was the second most effective extract, followed by the petroleum ether fraction of the plant.

In another study, (Mulla Wahid et al., 2011), found that the aqueous extracts of *A. indica* leaves were more active *in-Vitro* against *Entamoeba histolytica* (IC_50_ = 4.78 μg/ml) while the ethanol extracts of the plant leaves were more active against *Giardia intestinalis* compared to the standard amebicidal drug, emetine (IC_50_ = 0.99 μg/ml) and giardicidal drug, metronidazole (IC_50_ = 0.41 μg/ml). In contrast, Patil et al. (2012) found that the anthelmintic activity of the ethyl acetate fractions of *A. indica* Schott root was significant against *P. posthuma* compared to its alcohol extracts, although both extracts exhibited a dose-dependant manner of paralytic and death effects with a maximum concentration of 100 mg/ml. Meanwhile, Banik et al. (2014) found that the methanol extract of *A. macrorrhizos* root exhibited a dose-dependant anthelmintic activity against *P. Posthuma* with the most significant paralytic and death effects were recorded at a concentration of 80 mg/ml*.*

Based on the studies conducted, it was assumed that the anthelminthic and antiprotozoal activities of *A. indica* extracts and their fractions may be contributed by the presence of polyphenols and cyanogenetic glycosides (Mulla Wahid et al., 2010b; Mulla Wahid et al., 2011; Patil et al., 2012). Polyphenols are capable of binding to free proteins in the gastrointestinal tract of the host animal or glycoprotein on the cuticle of the parasite, leading to the death of the parasite (Patel et al., 2010). Nonetheless, the exact mechanism of action on how flavonoids and cyanogenetic glycosides exhibit anthelminthic property is still unclear (Mulla Wahid et al., 2010a; Patil et al., 2012).

### Antinociceptive and Anti-inflammatory Activity

The antinociceptive activity of ethanol extracts of *A. indica* leaves was investigated by (Mulla Wahid et al., 2010b) in albino rats using acetic acid-induced writhing response, hot plate, and tail-flick assays. It was found that the oral administration of the plant extract (200 and 400 mg/kg BW) protected the rats against both chemical- and thermal-induced noxious stimuli. The plant extract was able to significantly reduce the number of writhing induced by acetic acid, induced protection in rat tail immersion test, and increased the pain threshold of the rat in a hot plate assay. In addition, the anti-inflammatory activity of gels of the ethanol extract of *A. indica* leaves showed that the different gels of the plant extracts (5, 10, and 20%, respectively) produced a significant dose-dependant oedema inhibition in all the rat models (carrageenan- and formalin-induced paw oedema and arachidonic- and xylene-induced ear oedema) compared to the standards.

Acetic acid-induced writhing syndrome and causes analgesia by releasing prostaglandins, which then excite the pain nerve endings (Mulla Wahid et al., 2010a; Rahman, 2011). The ethanol extract of the plant leaves may show an analgesic effect *via* the inhibition of prostaglandin production. An oedema formation is a biphasic event in which the initial phase (within the first hour) is associated with the release of histamine and serotonin while the second phase is associated with the release of bradykinin and prostaglandin (Mulla Wahid et al., 2010b; Rahman, 2011). The potent antinociceptive and anti-inflammatory of *A. indica* extracts may be due to the presence of free radical scavenging bioactive molecules such as flavonoids, which target ROS and prostaglandins that were involved in the late phase of acute inflammation and pain perception (Mulla et al., 2010).

Further studies on the analgesic and anti-inflammatory activity of ethanol extract of *A. indica* tuber was carried out by Rahman (2011) in an acetic acid writhing model in mice and carrageenan-induced paw oedema in rat models, respectively. The extract produced approximately 41.33% and 68.375% writhing inhibition at doses between 300 and 600 mg/kg BW, respectively, which were comparable to the standard diclofenac sodium (79.08%) at 25 mg/kg BW. The extract also exhibited significant inhibitory effects on the formation of oedema between the first to the fifth hour of treatment. The highest inhibitory effects were observed at the third hour of treatment where the inhibition was 25.43% (300 mg/kg BW) and 41.05% (600 mg/kg BW) in mice and carrageenan-induced paw oedema in rat models, respectively. The findings were comparable to the standard aspirin (150 mg/kg BW).

The anti-inflammatory effects of isolated compounds from chloroform extract of *A. macrorrhiza* rhizome was conducted on LPS-induced NO production in RAW 264.7 cell lines. The viability of the cell was measured using the MTT method to determine the cytotoxic ability of the tested compounds to inhibit NO production. Despite the MTT results showing no obvious cytotoxicity on all the compounds against the RAW 264.7 cells at a concentration of 100 μM, all the compounds exhibited significant inhibitory effects on NO production with IC_50_ values ranged between 2.35 and 58.26 µM. The lignanamides (**26**–**30**) showed much stronger inhibitory effects than the monoindoles (**11**, **12**–**19**), which recorded similar IC_50_ values to that of indomethacin at 47.42 µM. The IC_50_ values of the three pairs of cis-trans isomers; **26** (13.33 µM) and **28** (17.79 µM), **27** (2.35 µM), and **29** (9.20 µM), strongly suggested that benzodihydrofuran-type lignanamides with a trans-configuration showed stronger inhibitory effects on NO production compared to those with a cis-configuration (Huang et al., 2017a).

### Brine Shrimp Lethality (Cytotoxic Activity)

Brine Shrimp Lethality Assay (BSLA) is a simple and low-cost bioassay technique to screen the toxicity of plant extracts and natural toxins. Cytotoxic compounds typically exhibit substantial activities in this assay (Islam et al., 2013; Banik et al., 2014; Hamidi et al., 2014; Haque et al., 2014), and is expressed as LC_50_ values which imply that extracts with a 50% concentration can kill the exposed population of brine shrimp. Theoretically, herbal extracts that exhibited LC_50_ < 1000 μg/ml are considered toxic, while extracts with LC_50_ < 1000 μg/ml are considered non-toxic (Hamidi et al., 2014). Since this bioassay has a good correlation with human solid tumour cell lines, the cytotoxic effects of plant extracts suggest that it can be applied for other cell line assay (Islam et al., 2013; Haque et al., 2014).

BSLA on the methanol extract of *A. macrorrhiza* Schott rhizome exhibited mild cytotoxicity effects against the brine shrimp nauplii with an LC_50_ value of 188.14 μg/ml compared to the standard drug vincristine sulphate at 11.32 μg/ml (Rahman et al., 2012). The results were similar to a previous study in which the ethanol extract of *A. indica* tuber exhibited a dose-dependant mortality rate against the brine shrimp nauplii with LC_50_ values of 81.09 μg/ml although the activity was substantially less compared to that of vincristine sulphate at 0.47 μg/ml (Islam et al., 2013).

The cytotoxic studies of different extracts of *A. fornicata* with vincristine sulphate as the standard drug exhibited moderate-to-potent cytotoxic activity ranging between 12.26 and 18.69 μg/ml against the brine shrimp nauplii. Moreover, ethanol extract of the root and ethyl acetate extract of the stolon recorded the most effective cytotoxic activity over all the different extracts tested and displayed potential anti-tumour activity (Haque et al., 2014).

Banik et al. (2014) also used the BSLA to monitor the cytotoxicity of methanol extracts of *A. macrorrhiza* roots and its different soluble fractions (petroleum ether, carbon tetrachloride, chloroform, and aqueous) with vincristine sulphate as the positive control. The results showed that all forms of the extracts possessed cytotoxic activities except for chloroform. The study suggested that the phytoconstituents in the plant extracts played an important role in cytotoxic activity even though the exact phytochemical compounds are yet to be discovered.

### Hepatoprotective Activity

The hepatoprotective activity of *Alocasia* species has been studied mostly using *A. indica* in carbon tetrachloride (CCl_4_)- and paracetamol (PCM)-induced liver-damaged rat models. The analysis is usually coupled with the use of Silymarin which acts as a positive control due to its known hepatoprotective effect (Mulla et al., 2009a). Both CCl_4_ and PCM or tylenol (acetaminophen) generate highly reactive free radicals that induce hepatocytes injury *via* lipid peroxidation. Consequently, an increase in the levels of liver marker enzymes (such as aspartic aminotransferase (AST), alanine aminotransferase (ALT), and ALP) and TBA Reactive Substances (TBARS) together with a decrease in total glutathione contents (GSH) were observed in both rat models (Mulla et al., 2009b; Patil et al., 2011; Pal et al., 2014a). The overconsumption of PCM produces a toxic reactive metabolite, N-acetyl-p-benzoquinone-imine (NAPQI), which will conjugate with glutathione, causing the depletion of glutathione and the increased hepatotoxicity level (Mulla et al., 2009a; Patil et al., 2011).

In early studies, (Mulla et al., 2009a), found that the hydroalcoholic extracts of *A. indica* (concentrations at 250 and 500 mg/kg BW) were capable of preserving the structural integrity of hepatocellular membrane in a dose-dependant manner in CCl_4_-and PCM-induced liver-damaged rat models, which were similar to that of Silymarin (100 mg/kg). The plant extracts were also able to reduce all the elevated levels of AST, ALT, and ALP to the respective normal levels. It was believed that the bioactive molecules that possessed free radical scavenging properties found in the plant extracts such as alocasin, polyphenolic compounds, and flavonoids contribute to the hepatoprotective effects of the plant. In addition, it was suggested that the plant extracts may interfere with the metabolism of cytochrome P450, thus preventing the formation of hepatotoxic free radicals.

The leaf juice of *A. macrorrhiza* at concentrations of 5 and 10 μL/ml exhibited an *in-Vitro* hepatoprotective effect on CCl_4_-and Tylenol-induced hepatocytes-damaged in rat liver slices. The extracts impaired the CCl_4_ and Tylenol mediated oxidative stress by decreasing the formation of free radicals and increasing hepatic glutathione levels by *de novo* synthesis of glutathione, its regulation, or both. Moreover, the leaf juice of *A. macrorrhiza* decreased the leakage of AST, ALT, and ALP from the rats’ liver slices into the surrounding medium. The possible mechanism of hepatoprotective activity by the *Alocasia* species could be due to superoxide scavenging activity by some of the constituents in the plant extracts, which reduced O_2_^·−^ to a non-radical form and removing oxygen from the reaction mixture (Patil et al., 2011).

Previously, the *in-Vivo* hepatoprotective effect of ethanol and aqueous extracts of *A. indica* (Roxb.) Schott tuber was investigated by Pal et al. (2014a) in CCl_4_-induced liver-damaged rats. The results showed that both extracts at a concentration of 200 mg/kg/day significantly reduced the level of AST and ALT by 65.32% and 77.36%, respectively compared to the group of CCl_4_-treated rats. Moreover, both extracts at similar concentration were capable of reducing a high level of malonaldehyde (MDA) (a hallmark of lipid peroxidation in the ethanol-attenuated liver) and increased the level of GSH by 41.39% and 55.46%, respectively. In addition, the histological characteristics of the damaged hepatocytes were recovered with a significant absence of fat droplets and normal patterns of central vein and cell plates of the hepatocytes. SOD and catalase enzyme activity were also detected in both plant extracts, suggesting that the hepatoprotective effect of the extracts may be contributed by the antioxidant property of the bioactive molecules that were present in the extracts such as phytosterols, alkaloids, flavonoids, and tannins.

The ethanol extract of *A. indica* (Roxb.) Schott exhibited hepatoprotective activity with a significant effect recorded at a concentration of 400 mg/kg. It was believed that the flavonoids and phenolic compounds present in the plant extracts could have stabilised and repaired the hepatocyte membrane, recovered the level of biomarker enzymes, and enhanced the antioxidant enzymes (SOD and catalase), which were drastically decreased by the alcohol. The plant extracts also assisted in down-regulating the NF-κB signal in ethanol-induced injury by suppressing the NF-κB dependant target genes expression on Kupffer cells in the alcohol-treated liver. Moreover, the plant extracts were able to reduce the expression of caspase-3 in the alcohol-induced rat which supported the antiapoptotic action of the extract (Pal et al., 2014b).

In addition, the hepatoprotective effect of the *Alocasia* species in alcohol-induced liver-damaged rat models was studied (Pal et al., 2014b). Based on the results, it was revealed that excessive alcohol consumption enhances NADH production which in turn leading to more production of fatty acids and triglycerides. High alcohol intake can also contribute to the leakage of ALT and AST into the plasma as well as leakage of γGT into the blood. MDA and NO substantially increased the ethanol-attenuated liver while GSH, SOD, and catalase were significantly reduced after the mice were intoxicated with ethanol (Pal et al., 2014b).

### Anti-Hemagglutinin Activity

The anti-hemagglutinin properties of lectins isolated using affinity chromatography on asialofetuin-linked amino activated beads from *A. cucullata* and *A. indica* have been tested (Singh et al., 1993; Kaur et al., 2005). Kaur et al. (2005) investigated the anti-hemagglutinin activity of N-acetyl-D-lactosamine (LacNAc) (**43**), isolated from *A. cucullata* tuber on erythrocytes and lymphocytes of rabbit, guinea-pig, sheep, goat, and human. The study found that the lectin agglutinated normal erythrocytes in rabbit and guinea-pig, and human lymphocytes while the lectin agglutinated sheep lymphocytes only after the neuraminidase treatment. The Minimal Erythrocytes Agglutinating Protein Concentration (MEAPC) of the lectin was reduced 8 times following neuraminidase treatment of rabbit erythrocytes.

Besides, (Singh et al., 1993), found that the *A. indica* lectin could agglutinate normal Red Blood Cells (RBCs) in rabbit and guinea-pig and neuraminidase-treated rat erythrocytes but was inactive against human ABO erythrocytes. Based on these results, the ability to agglutinate lymphocytes was largely confined to neuraminidase-treated cells. The neuraminidase treatment allowed better access to receptors by removing terminal sialic acid groups, thus exposing the lectin receptors and decreased the net negative charge on the cell surface (Kaur et al., 2005).

### Anti-Constipation and Diuretic Activities

The leaves and rhizomes of *A. macrorrhiza* are traditionally used to treat constipation, digestion, laxative, and diuretic (Srivastava et al., 2012). In order to understand the anti-constipation and diuretic activities of *Alocasia* species, Mubeen et al. (2012) conducted an *in-Vivo* study on the laxative and diuretics effects of ethanol extract of *A. macrorrhiza* leave (100, 200, and 400 mg/kg) in Wister albino rats. The laxative study was carried out in a rat model with low-fibre diet-induced constipation while agar-agar (300 mg/kg p.o) was used as a positive control. Based on the result, doses lower than 100 mg/kg failed to show the laxative effect. On the contrary, doses at 200 and 400 mg/kg significantly increased the faecal output of rats and showed a dose-dependant increase in faecal output of rats when compared to the control group. Nonetheless, the laxative activity demonstrated at a maximum dose of 400 mg/kg was significantly lesser than the standard agar-agar. The results indicated that *A. macrorrhiza* ameliorated low-fibre diet-induced constipation in rats, therefore indicating the suitability for human patients suffering from constipation due to their diet style.

In addition, the diuretic activity was conducted using the Lipschitz test with furosemide (20 mg/kg p.o.) used as the positive control. The preliminary phytochemical test revealed the presence of flavonoid, cholesterol, amino acids, glycoside, and alkaloid in the ethanolic extract of *A. macrorrhiza* (Mubeen et al., 2012). The results showed that the ethanolic extract increased urinary output and urinary ion concentration of the rats at higher doses (400 mg/kg BW) but was ineffective at a lower dose of 100 mg/kg. Although a significant increase in the excretion of Na^+^, K^+^, and Cl^−^ was found at a dose of 400 mg/kg, the diuretic activity was significantly less than that of the standard drug furosemide. The increase in the ratio of the concentration of the excreted Na^+^ and K^+^ ions indicated that the ethanolic extract increased the Na^+^ ion excretion to a greater extent than the K^+^ ion, which is an essential requirement for an ideal diuretic with minimised hyperkalemic side effect.

### Radioprotective Activity

The ethanolic extract of *A. indica* tuber was active in radioprotective activity. The rats were fed with the ethanol extract for 7 days, before being irradiated with gamma rays at a dose of 2.9 Gy for 24 h. Finally, the rats were dislocated to analyse the effect of radiation on the uterine and ovarian organs through several parameter measurements. The result indicated that *A. indica* exhibited strong radioprotective activity to prevent female infertility by increasing fertility status, reducing the ROS level of granulosa cells with increasing granulosa cell viability and steroidogenic enzyme activity. (Prasad et al., 2019).

### Acute Toxicity Study

In acute toxicity study of *A. indica* showed no mortality and no signs of toxicity after the administration of a limit dose of 2000 mg/kg BW of the extract. Therefore, 1/10^th^ of the dose was prescribed as the safe and effective dose. In addition, no mortality was recorded at the maximum dose (up to 1000 mg/kg body weight) of ethanol extract of *A. indica* tuber after an observation period of 48 h in mice. The estimated minimum lethal dose of the extract was more than 1000 mg/kg body weight (Islam et al., 2013). Furthermore, hydroalcoholic extracts of *A. indica* leaves did not result in mortality up to a dose of 2000 mg/kg p.o. (Mulla et al., 2009b). Peng et al. (2013) confirmed that the toxicology measurement on gavage feeding of aqueous extract of *A. cucullata* root at a concentration of 16 g/kg BW was safe and harmless (Patil et al., 2012).

## Conclusion

In conclusion, the medical application of *Alocasia* species has been proven in many *in-Vitro* and *in-Vivo* studies in which the biological activities of the plants extract were associated with the presence of phytochemicals, mainly flavonoids, alkaloids, and phenolic compounds. Of the many *Alocasia* species, the most predominantly studied for drug development are the *A. macrorrhiza* (L.) G. Don and *A. indica* Schott, which among others have demonstrated anti-cancer, antioxidant, and antimicrobial activities. Given that the use of specific isolated compounds from the plant extracts in biological studies are currently limited, further studies using these compounds could help to understand the mechanism and treatment efficiency, especially towards cancer treatment. Furthermore, new findings and discovery of the vast potential application of *Alocasia* species would provide more efficient therapy with a new mechanism of action, reducing the adverse effects of anti-cancer drugs, and encourage the development of new anti-cancer drugs. The genus *Alocasia*, which is found scattered in Asia, Southeast Asia, and Australia, has been traditionally used to treat various diseases and represent enormous diversity worldwide, but not much has been explored yet. With current technological developments, it is hoped that bioactive compounds that can effectively inhibit cancer cells can be identified and developed.

## Data Availability

The original contributions presented in the study are included in the article/Supplementary Material, further inquiries can be directed to the corresponding authors.
